# Amelioration of muscle and nerve pathology of Lama2-related dystrophy by AAV9-laminin-**α**LN linker protein

**DOI:** 10.1172/jci.insight.158397

**Published:** 2022-07-08

**Authors:** Karen K. McKee, Peter D. Yurchenco

**Affiliations:** Department of Pathology and Laboratory Medicine, Rutgers University — Robert W. Johnson Medical School, Piscataway, New Jersey, USA.

**Keywords:** Muscle Biology, Neuroscience, Extracellular matrix, Laminin

## Abstract

LAMA2 deficiency, resulting from a defective or absent laminin α2 subunit, is a common cause of congenital muscular dystrophy. It is characterized by muscle weakness from myofiber degeneration and neuropathy from Schwann cell amyelination. Previously it was shown that transgenic muscle-specific expression of αLNNd, a laminin γ1–binding linker protein that enables polymerization in defective laminins, selectively ameliorates the muscle abnormality in mouse disease models. Here, adeno-associated virus was used to deliver linker mini-genes to dystrophic *dy^2J^/dy^2J^* mice for expression of αLNNd in muscle, or αLNNdΔG2′, a shortened linker, in muscle, nerve, and other tissues. Linker and laminin α2 levels were higher in αLNNdΔG2′-treated mice. Both αLNNd- and αLNNdΔG2′-treated mice exhibited increased forelimb grip strength. Further, αLNNdΔG2′-treated mice achieved hind limb and all-limb grip strength levels approaching those of WT mice as well as ablation of hind limb paresis and contractures. This was accompanied by restoration of sciatic nerve axonal envelopment and myelination. Improvement of muscle histology was evident in the muscle-specific αLNNd-expressing mice but more extensive in the αLNNdΔG2′-expressing mice. The results reveal that an αLN linker mini-gene, driven by a ubiquitous promoter, is superior to muscle-specific delivery because of its higher expression that extends to the peripheral nerve. These studies support a potentially novel approach of somatic gene therapy.

## Graphical abstract

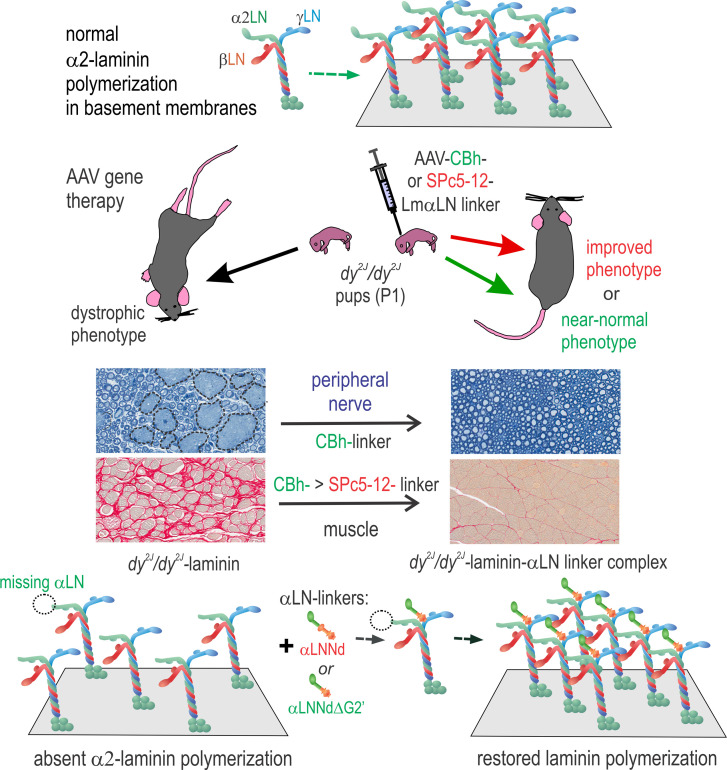

## Introduction

The laminin α2 subunit is expressed in the basement membranes (BMs) of myofibers and peripheral nerve Schwann cells (SCs) (1). During assembly, laminin α2 heterotrimers (α2β1γ1, α2β2γ1 subunits) adhere to cognate cell surfaces to assemble a primary scaffold that matures by binding to agrins, nidogens, and type IV collagen, with the last forming a covalently cross-linked network (2). While the C-terminal LG domains of α2-laminins bind to α-dystroglycan and α_7_β_1_ integrin cell surface receptors, the LN domains of the N-terminal 3 short arms of adjacent heterotrimeric laminins bind to each other to create a sheet-like polymer (3).

Mutations in the *LAMA2* gene that result in either absent or defective laminin α2 subunits cause an autosomal recessive congenital muscular dystrophy (1). The muscle component of the disease is characterized by weakness resulting from progressive muscle degeneration, myofiber death, satellite cell–mediated regeneration, chronic inflammation, and fibrosis. Patients have also been found to develop a peripheral neuropathy arising (based largely on mouse studies) from SC axonal amyelination (4). Most cases of the dystrophy/neuropathy arise from mutations that result in a complete or near-complete loss of the α2 subunit. Patients with these mutations rarely ambulate and typically die prematurely of muscle wasting and respiratory failure. A subset of LAMA2 mutations cause a milder form of the disease. These are often missense mutations at the 5′ end of the LAMA2 gene. Several α2-LN mutation loci are located in the LN domain, are highly conserved within the laminin family, and are likely to interfere with polymerization (5). Collectively, disease resulting from laminin α2 deficiency is now referred to as LAMA2-related dystrophies (LAMA2-RDs) (6).

**Figure 1:**
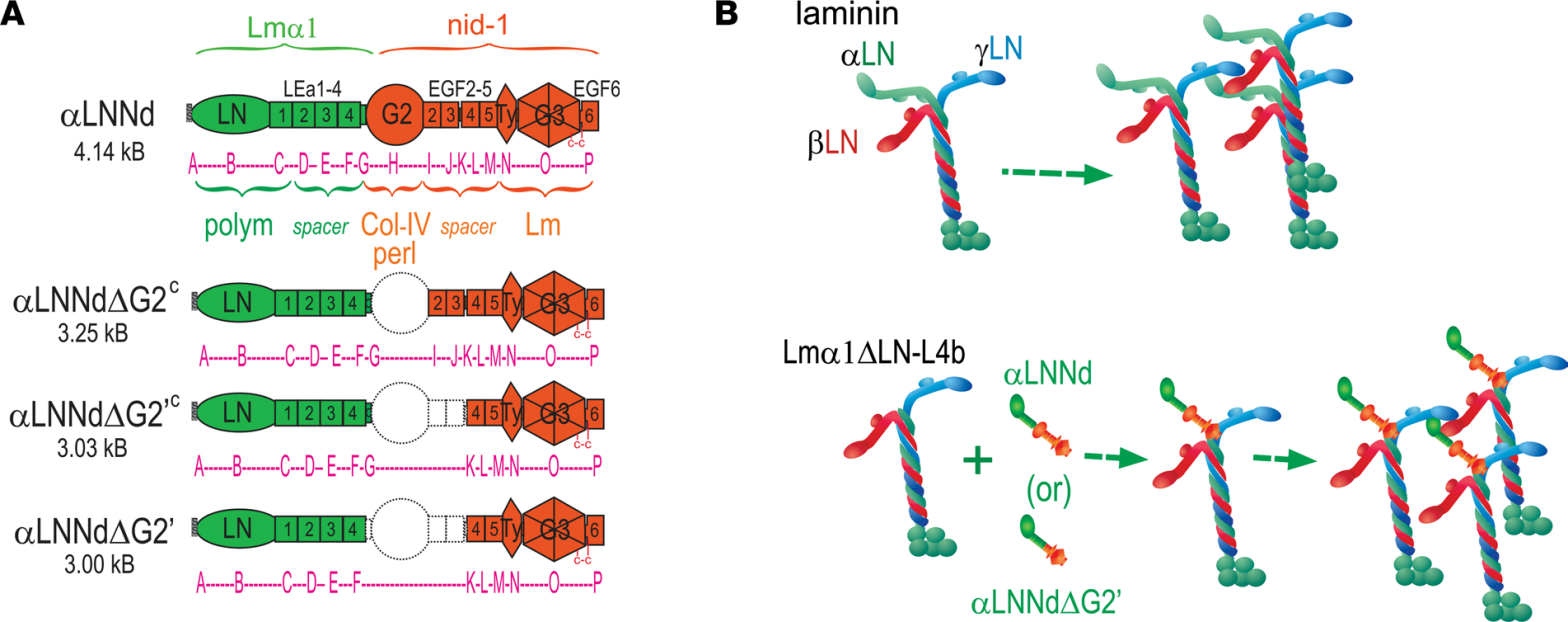
Laminin-binding linker proteins. (**A**) Protein domains of full-length LNNd and shortened versions lacking the G2 domain (laminin domains in green and nidogen-1 domains in orange). These are lettered as follows: A, signal sequence; B, laminin (Lm) α1LN; C–F, LEa1–LEa4; G, L4 segment and nidogen EGF-1; H, G2 (consists of EGF plus β-barrel); I-M, EGF-type domains 2–5 with intervening spacer; N, thyroglobulin (Ty) domain; O, G3 propeller; P, terminal EGF-6. The Ty-G3 propeller-EGF6 domain complex mediates laminin binding. (**B**) Linker protein-dependent laminin polymerization. Lm1ΔLN-L4b is a modified Lm111 in which the α1LN domain along with most of the α1 short arm has been deleted, rendering it unable to polymerize. Polymerization is enabled following binding of a suitable linker protein to the laminin via its nidogen-binding locus.

Although a variety of therapeutic modalities hold promise, none so far have been demonstrated to be effective in the clinic (1). Some of the approaches treat the inflammatory, apoptotic, and fibrotic sequelae of the dystrophy (7–9). Others seek to correct the underlying structural defect in the BM by delivering a substitute laminin or by altering surviving and/or compensating laminins with small laminin-binding proteins (1, 10–13). One of these laminin-binding proteins is αLNNd, a chimeric protein consisting of the α1LN and 4 adjacent LE domains fused to the G2 through G3 domains of nidogen-1 (14). The G3 domain allows for a high-affinity attachment to laminins, creating an artificial short polymerization arm tipped with the α1-LN domain. The G2 domain binds to type IV collagen and to the heparan sulfate proteoglycan, perlecan (Figure 1).

There are several mouse models of Lama2 deficiency that approximate the range of clinical severity in humans (1). Two of these (*dy^W^*/*dy^W^* and *dy^3K^*/*dy^3K^*, the latter corresponding to the complete *Lama2* knockout) exhibit severe disease with a shortened life span. Another, *dy^2J^*/*dy^2J^*, is a model for the ambulatory form of the disease. It is caused by a splice donor mutation that results in an N-terminal in-frame deletion with α2LN domain degradation (15–17). Muscle histology shows fibrosis, chronic inflammation, and regeneration. All the mouse models are accompanied by sciatic nerve amyelination (18). Examination of the dystrophic sciatic nerve reveals compact groups of naked axons. The defect is a developmental one of inhibited radial axonal sorting, the process by which SC precursors envelop, sort, and then myelinate axons (19). The process occurs during the first 2 weeks of murine postnatal life (18). While muscle-specific transgene expression of αLNNd in the *dy^2J^*/*dy^2J^* mouse muscle was found to substantially ameliorate forelimb muscle strength, hind limb strength was minimally improved, reflecting a failure to correct the sciatic nerve paresis (12).

Adeno-associated virus (AAV) is a promising somatic gene delivery system for therapy in which high expression can be achieved in target tissues (20). Although the newly synthesized protein levels have been reported to be reduced in treated patients due to immune responses to transgene products and AAV capsid (21), this problem has been addressed by avoiding the creation of transgene neo-antigen, optimizing serotype, and adding immunosuppressive therapy (22, 23). A limitation of AAV-mediated therapy is the small genomic carrying capacity of the capsid. Of relevance to the current study, the open reading frame of the DNA coding for αLNNd (4.14 kb) approaches the AAV capsid capacity (~5 kb, with a desired practical limit of about 4.7 kb, the size of the native DNA in AAV). The remaining capacity of ~ 0.9 kb allows for only a small simple promoter without enhancing elements and poly-A tail. One identified short promoter that can accommodate full-length αLNNd, reported to provide a 6- to 8-fold increase in activity over the CMV promoter, is the muscle-specific SPc5-12 (24, 25). Other promoters likely to mediate higher expression in desired tissues, particularly those accompanied by enhancing or regulatory sequences, are too large unless the linker protein and corresponding DNA can be substantially reduced in size.

In the current study we describe the redesign of the linker protein αLNNd to create a smaller linker, αLNNdΔG2′, for DNA insertion into AAV; a comparison of AAV-driven expression of αLNNd and αLNNdΔG2′ with muscle-specific and universal promoters; and an analysis of mouse behavior with comparisons of muscle and peripheral nerve histology. We show that, compared with a muscle-specific promoter driving αLNNd, employment of the ubiquitously expressing CBh promoter accompanied by a posttranslational regulatory element driving a shortened version of αLNNd achieves greater muscle benefit along with prevention of amyelination.

## Results

### Linker protein design and characterization.

Various size reduction strategies were considered to enable AAV-mediated linker expression. αLNNd possesses a linear array of domains that provide an N-terminal polymerization domain, a central type IV collagen– and perlecan-binding G2 domain, and a C-terminal laminin-binding G3 domain complex (Figure 1). A reduction approach chosen was to delete the large internal G2 and distal EGF domains with a comparison of polymerization and matrix assembly on cultured SCs and myotubes prior to initiation of mouse AAV injections.

The total WT viral genome insert size for AAV is 4.7 kb. However, sizes of 5 kb and even greater have been accommodated, albeit with a lower efficiency of full DNA incorporation (26). While there are promoters small enough to be used with full-length αLNNd, the options for achieving high expression are limited, particularly if one wishes to add enhancing or stabilizing elements. Further, given the muscle and peripheral nerve (and brain) involvement in laminin-deficient dystrophy, it was thought that a universal promoter would be preferable to a muscle-specific promoter. A given limitation for any reduction strategy for αLNNd is that the LN polymerization domain, distal fused LEa1 domain, laminin-binding G3 laminin-binding complex must be maintained to achieve the desired linker function and affinity. That left the intervening LE, G2, and nidogen EGF-like domains for consideration. Of these, elimination of G2 (843 bp) reduced the open reading frame from 4.14 to 3.30 kb (Figure 1). A further reduction was obtained by removal of a half-LE (33 bp) and 2 EGF-like domains (126 × 2 bp) immediately distal to G2 for a final size of 3.01 kb. Recombinant linker proteins were generated and coupled to nonpolymerizing Lmα1ΔLN-LEa and analyzed for self-assembly in vitro (Supplemental Figure 1; supplemental material available online with this article; https://doi.org/10.1172/jci.insight.158397DS1) to determine if the protein modifications reduced efficiency.

The concentration dependency of polymerization for all the protein complexes (Supplemental Figure 1A) was found to be similar to WT laminin 111 (Supplemental Figure 1B), with the complexes assembling equivalently on high-passage SCs (Supplemental Figure 1, C–E) (14, 27). Previous studies revealed that full-length αLNNd coupled to nonpolymerizing laminins on SCs and C2C12 myotubes improves laminin self-assembly while also permitting collagen IV assembly (12, 14, 27). Loss of G2 was expected to prevent linker binding to collagen IV and perlecan, an expectation illustrated on SC and myotube coincubation with Lmα1ΔLN-LEa linker complexes in the presence of collagen IV or perlecan (Supplemental Figure 2). However, the presence of nidogen-1 in the mixture, approximating the nidogen-1 that is endogenously expressed in nearly all tissues, restored collagen IV and perlecan coassembly (Supplemental Figure 2, A–G). An optimal balance between increased laminin accumulation and increased collagen IV or perlecan accumulation was achieved when linker lacking G2 was maintained equimolar to nidogen. This relationship likely reflects the competition of the 2 proteins for the identical binding site of the Lmγ1 subunit (12, 14). Since nidogens are normally expressed in *dy^2J^/dy^2J^* mice that lack a polymerizing α2 laminin, the expectation was that deletion of G2 from αLNNd would not adversely affect collagen IV and perlecan assembly.

Laminin polymerization has been found to play a role in axonal myelination in dorsal root ganglion (DRG) cultures, an in vitro model for peripheral nerve development (18). Of note, full-length αLNNd coupled to Lmα1ΔLN-L4b increased myelination, unlike Lmα1ΔLN-L4b alone (28). To determine if G2 deletion would affect myelination, Lmα1ΔLN-L4b was incubated with αLNNdΔG2′ (Supplemental Figure 2, H and I). The preparation containing the shortened linker increased myelination to levels similar to that of WT Lm111.

### AAV-treated dystrophic mouse ambulation and grip strength.

Laminin-binding linker single-stranded DNA was delivered to newborn pups with AAV9 vectors. The component parts of the somatic genes are listed in Supplemental Table 1. αLNNd (4.1 kb) used the small muscle-specific promoter SPc5-12 (24) while αLNNdΔG2′ (3.0 kb) used the ubiquitous CBh promoter consisting of the β-actin promoter and CMV enhancer (29) as well as the woodchuck hepatitis virus posttranscriptional regulatory element (WPRE) that is reported to enhance expression when delivered by viral vectors (30, 31). Virus was injected into 1-day-old dystrophic pups via the temporal vein with the following doses: AAV9-SPc5-12-αLNNd at 3.8 × 10^11^ vector genomes/gram (vg/g) and AAV9-CBh-αLNNdΔG2′ at 4.2 × 10^11^ vg/g (high) and 1.3 × 10^11^ vg/g (low). One mouse received AAV9-CBh-αLNNdΔG2′ at 0.7 × 10^11^ vg/g.

Dystrophic (*dy^2J^/dy^2J^*) mice exhibited the characteristic hind limb retraction phenotype when lifted by the tail at about 3 weeks of age. In contrast, neither WT (*dy^2J^/+*, *+/+*) nor AAV9-CBh-αLNNdΔG2′–treated *dy^2J^/dy^2J^* mice (both high and low dose) exhibited this phenotype at 3 weeks or over the next 12 weeks (Table 1). By about 7 weeks of age, the untreated dystrophic mice developed hind limb contractures seen as a continual rigid extension of one or both hind limbs. This altered gait and impeded ambulation. The AAV9-SPc5-12-αLNNd–treated dystrophic mice also developed hind limb weakness and contractures with slightly less severity compared with untreated mice. In contrast, the ambulation of both WT and AAV9-CBh-αLNNdΔG2′–treated dystrophic mice (high and low dose, total of 17 mice) appeared normal and could not be distinguished from each other (Table 1, Supplemental Figures 3 and 4, and Supplemental Videos 1–3). Tracings of ambulatory activity and frequency of grid-barrier crossings revealed that the CBh-αLNNdΔG2′–treated dystrophic mice (low dose) increased to levels similar to the WT mice (Supplemental Figure 4). One female/male pair of dystrophic mice that had been treated with high-dose AAV9-CBh-αLNNdΔG2′ was kept together for 11 months of age after completion of the grip strength analysis. The pair produced *dy^2J^/dy^2J^* offspring in 2 litters. The offspring of the CBh-αLNNdΔG2′–treated *dy^2J^/dy^2J^* mice, all confirmed as *dy^2J^/dy^2J^*, exhibited the same dystrophic ambulation as observed in other untreated *dy^2J^/dy^2J^* mice.

**Figure 2:**
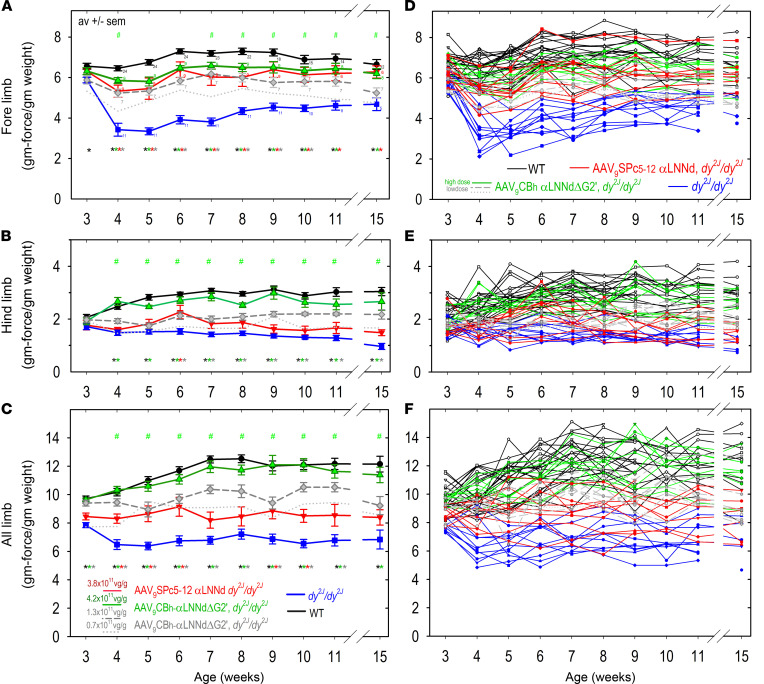
Mouse grip strength. Specific grip strength values (average, SEM; **A**–**C**) and corresponding individual mouse values (**D**–**F**) are shown for forelimbs (**A** and **D**), hind limbs (**B** and **E**), and all limbs (**C** and **F**). *dy^2J^/dy^2J^* pups at postnatal day 1 were injected with AAV9-SPc5-12-αLNNd (red solid line and red inverted triangles; 5.5 × 10^11^ vg/pup) or with AAV9-CBh-αLNNdΔG2′ at 6.0 × 10^11^ vg/pup (green solid line; open green triangles), at 1.9 × 10^11^ vg/pup (gray short dashed line, gray diamonds), and a single mouse set at 0.95 × 10^11^ vg/pup (gray dotted line) via the temporal vein (*n* = 7–9 mice) and compared with untreated WT (black solid line, black circles) and *dy^2J^/dy^2J^* (blue solid line, blue squares) mice from 3 to 15 weeks of age. Statistical significance was determined from the average and SEM by 1-way ANOVA followed by Holm-Šidák test pairwise comparisons. The full set of 1-way ANOVA values are shown in Supplemental Table 2. Green hashtag symbols above the plots indicate no significant difference was found between WT and AAV9-CBh-αLNNdΔG2′ high-dose-treated *dy^2J^/dy^2J^* mice. Colored asterisks below plots indicate a significant difference between untreated *dy^2J^/dy^2J^* mice compared to WT (black), higher-dose AAV9-CBh-αLNNdΔG2′ (green), AAV9-CBh-αLNNdΔG2′ *dy^2J^/dy^2J^* (red), and/or lower-dose AAV9-CBh-αLNNdΔG2′ *dy^2J^/dy^2J^* (gray) mice.

Specific forelimb, hind limb, and combined limb (all-limb) grip strengths (grams force/grams mouse weight) were measured in control WT, dystrophic, and treated dystrophic mice (Figure 2; plots of individual mouse grip strengths as a function of age shown in Supplemental Figure 4 with 1-way ANOVA in Supplemental Table 2). At 3 weeks, very little reduction of grip strength was detected in the forelimbs of dystrophic mice, whereas modest differences were noted in the hind limbs and combined forelimbs and hind limbs. By 4 weeks, the specific forelimb grip strength was considerably reduced in untreated dystrophic mice. Muscle-specific αLNNd-treated dystrophic mouse forelimb grip strengths were found to lie between the dystrophic and WT values. Forelimb grip strength values of ubiquitously expressed αLNNdΔG2′-treated (high dose) dystrophic mice were similar to muscle-specific values and nearly as high as WT levels. Hindlimb and all-limb grip strength values for ubiquitously expressed αLNNdΔG2′-treated (high dose) dystrophic mice were also similar to WT values. However, muscle-specific αLNNd-treated dystrophic hind limb and all-limb grip strength values were considerably less than WT values. Dystrophic mice injected with a low dose of αLNNdΔG2′ virus (and a single mouse with a very low dose) exhibited elevated forelimb, hind limb, and all-limb levels but somewhat less than the high-dose-treated mice. The low-dose-treated mice retained a normal type of ambulation and normal outward extension of hind limbs when suspended by their tails and could not be distinguished from the high-dose-treated dystrophic mice by inspection.

### Linker protein tissue immunostaining.

**Figure 3:**
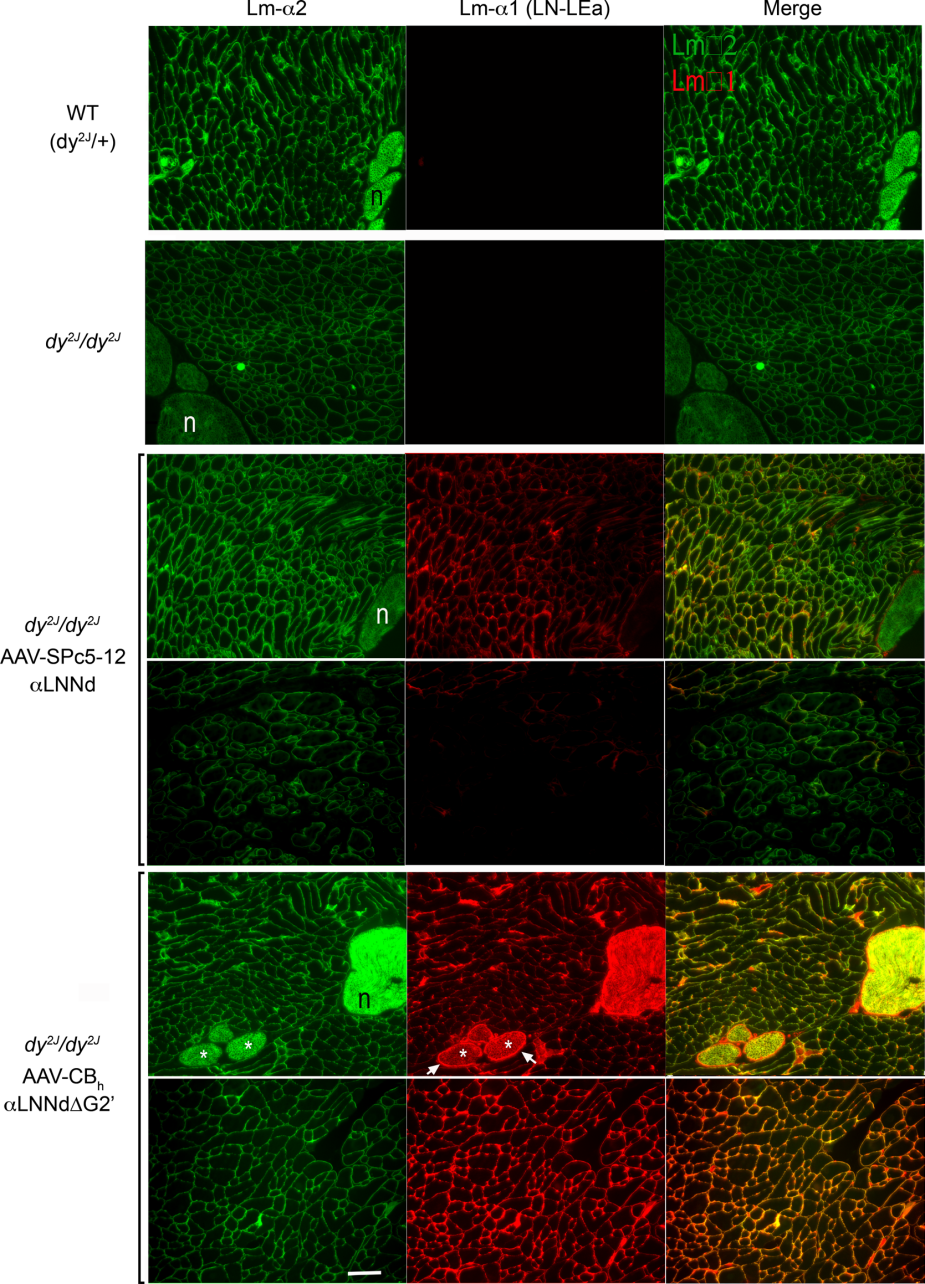
Laminin immunofluorescence in skeletal muscle. Frozen sections from lower limb muscle were immunostained to detect laminin α2 and α1 (N-terminal moiety marker for linker protein expression). Muscle was obtained from WT (*dy^2J^/+*), dystrophic (*dy^2J^/dy^2J^*), and AAV-treated dystrophic mice at 9 weeks of age. Laminin α2 was detected in both WT and dystrophic muscle. Two fields are shown for each linker-treated condition to show the range of variability of immunofluorescence intensity. Linker protein (αLNNd and αLNNdΔG2′) was detected only in AAV-treated muscle. Peripheral nerve branches (labeled “n”) were detected with the laminin α1 epitope only in mice treated with AAV-CBh-LNNdΔG2′. (Bar, 100 μm.) Lmα2 epitope was confined to endoneurium (asterisks) while Lmα1 linker epitope was present in both endoneurium and perineurium (arrows).

αLNNd and αLNNdΔG2′ linker protein expression was examined by immunofluorescence microscopy in limb skeletal muscle and sciatic nerve at 9 weeks age. Linker protein was detected in muscle in both AAV-treated mouse groups but only in peripheral nerve in the AAV9-CBh-αLNNdΔG2′–treated mice (Figure 3). Laminin α2 was present in the sarcolemma, vessel walls, and peripheral nerve in WT, slightly to moderately reduced in muscle BM, and considerably reduced in peripheral nerve BM in untreated dystrophic muscle. Muscle laminin α2 was noted to be increased in most regions of AAV9-SPc5-12-αLNNd–treated dystrophic muscle and all regions of AAV9-CBh-αLNNdΔG2′–treated muscle and peripheral nerve. However, there were focal regions of AAV9-SPc5-12-αLNNd–treated muscle that exhibited prominent dystrophic features (rounded and variable sized myofibers). These regions stained weakly for αLNNd linker protein and Lmα2 subunit. Compared with muscle-specific expression, Lmα2 and Lmα1LN immunofluorescence was brighter and distributed more evenly. Collagen IV and perlecan immunostaining was similar in WT, dystrophic, and AAV-treated dystrophic mice (Supplemental Figure 5).

### Sciatic nerve.

Myelination of peripheral nerves through radial axonal sorting occurs as a late developmental process in which SC precursors envelop small groups of naked axons with pseudopod-like extensions, followed by a sorting process to achieve a 1:1 SC/axon ratio with myelination of axons with a diameter of more than 1 μm (32, 33). The small-caliber axons (nearly all ≤1 μm diameter) within the SCs are enveloped with single membranes of SCs that are plasma membrane delineated and BM coated (“Remak bundles”). It has been previously reported that *dy^2J^/dy^2J^* and other laminin α2–deficient mice fail to develop peripheral nerve myelination during the perinatal period (33, 34), with laminin-dependent myelination recapitulated in DRG cultures (28).

**Figure 4:**
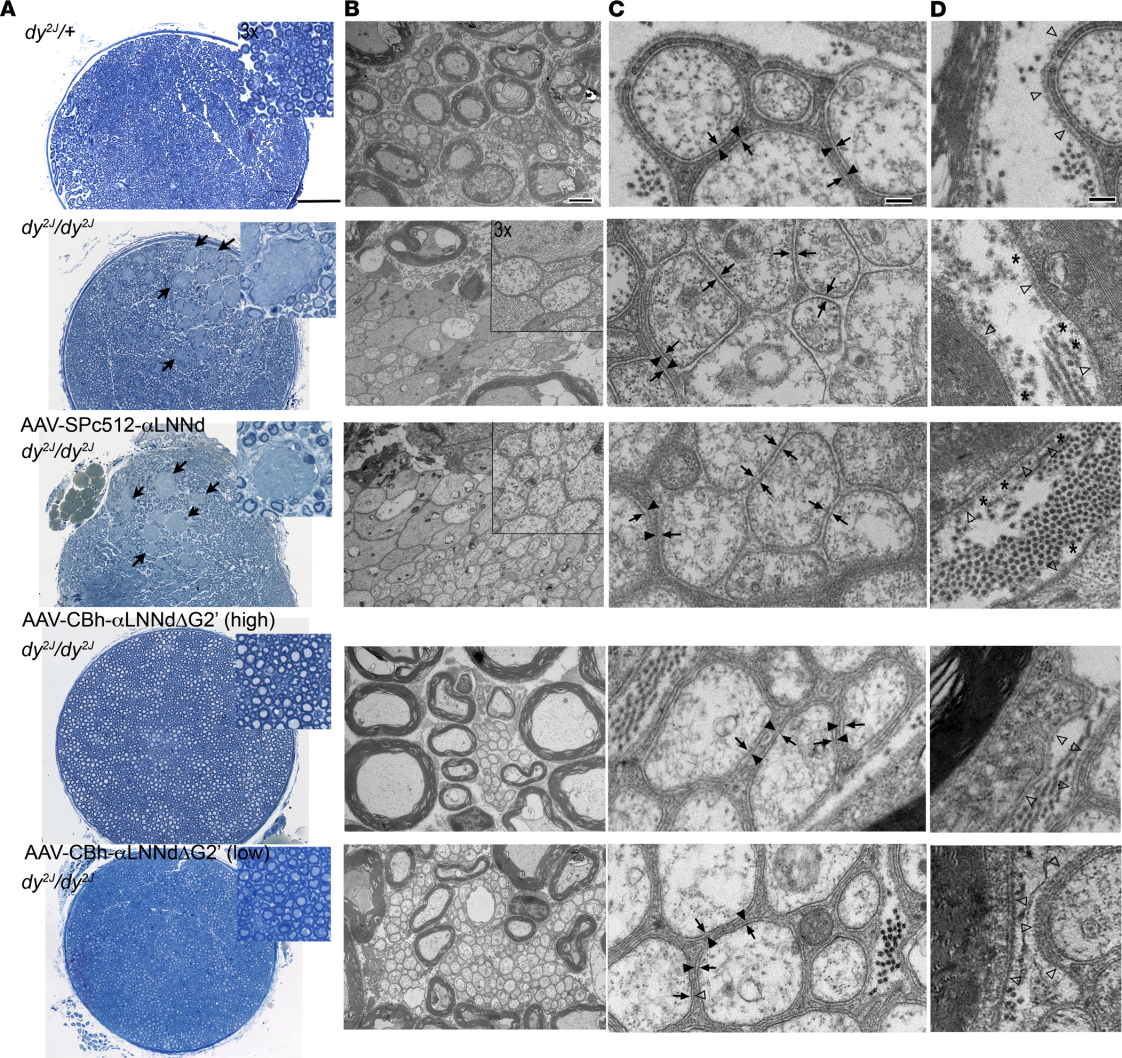
Sciatic nerve morphology. (**A**) Representative methylene blue–stained semithin sections. Amyelination patches (examples indicated by arrows) are present in the sciatic nerves of *dy^2J^/dy^2J^* and AAV-SPc5-12-αLNNd–treated *dy^2J^/dy^2J^* mice, but not in WT (*dy^2J^/+*) or AAV-CBh-αLNNdΔG2′ (high and low dose) treated *dy^2J^/dy^2J^* mice. Bar = 100 μm. (**B**) Electron micrographs. Myelinated axons adjacent to normal-appearing Remak bundles (enveloped small-caliber axons) are present in the nerves of *dy^2J^/+* and AAV-CBh-αLNNdΔG2′–treated *dy^2J^/dy^2J^* mice. Bar, 2 μm. (**C**) Higher magnification views of Remak bundles (bar, 200 nm) reveal that the axons of the *dy^2J^/+* and AAV-CBh-αLNNdΔG2′–treated *dy^2J^/dy^2J^* Remak bundles each have an inner axonal membrane (skinny arrow) enveloped by a SC membrane (solid arrowhead). Remak bundles of *dy^2J^/dy^2J^* and AAV-SPc5-12-αLNNd–treated *dy^2J^/dy^2J^* contain a mixture of enveloped and naked axons. (**D**) SC BMs (empty arrowheads) are continuous in *dy^2J^/+* and AAV-CBh-αLNNdΔG2′–treated *dy^2J^/dy^2J^* and discontinuous (asterisks) in untreated and AAV-SPc5-12-αLNNd–treated *dy^2J^/dy^2J^* nerve.

**Figure 5:**
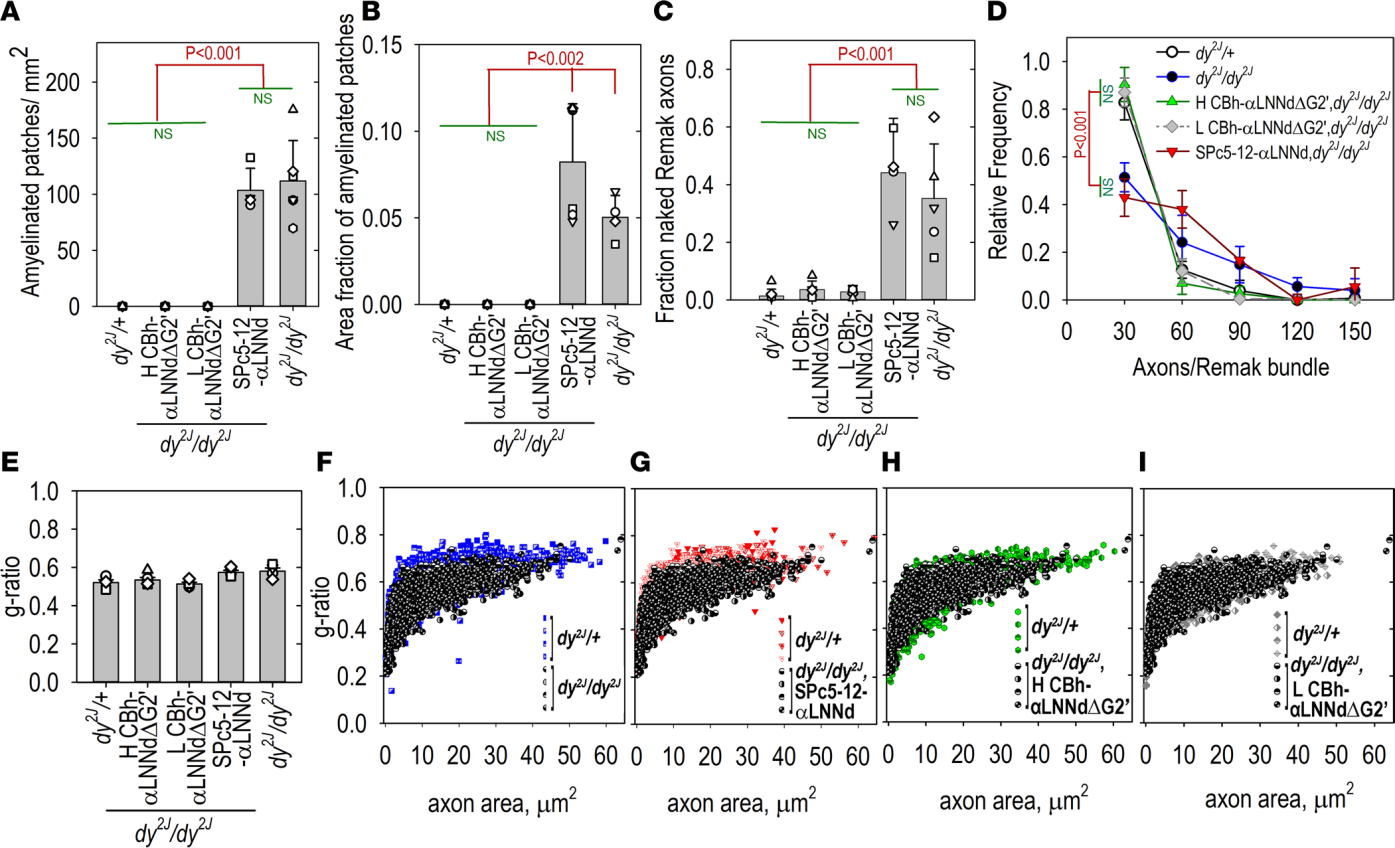
Sciatic nerve morphometry. Methylene blue–stained semithin and electron micrographs of proximal sciatic nerve from 15-week-old mice (4 to 6 mice/condition) were analyzed for (**A**) the number of amyelination patches per unit cross-sectional area (av. ± SD), (**B**) the fractional area of occupancy of amyelination patches, (**C**) the fraction of nonenveloped (naked) axons/total number of axons in Remak bundles (av. ± SD), (**D**) the total number of axons/Remak bundle, (**E**) overall g-ratios (av. ± SEM), and (**F**–**I**) *dy^2J^/+* pairwise plots of individual myelinated axon g-ratios versus axon area for untreated and treated *dy^2J^/dy^2J^*. *dy^2J^/dy^2J^* sciatic nerves, unlike WT *dy^2J^/+* nerves, contained multiple amyelination patches of tightly packed naked axons and substantial fractions of nonenveloped (naked) axons with enlarged Remak bundles. Sciatic nerves from *dy^2J^/dy^2J^* mice treated with either high (H) or low (L) doses of AAV-CBh-αLNNdΔG2′ were completely devoid of amyelination patches and Remak bundles. Statistical significance (**A**–**E**) was determined from the average and SD by 1-way ANOVA followed by Holm-Šidák test pairwise comparisons. Myelin thickness (inverse of g-ratios) for combined axonal areas are not statistically different for *dy^2J^/+* and *dy^2J^/dy^2J^* nerves.

The WT sciatic nerves of 15-week-old mice revealed a normal cross-sectional distribution of myelinated axons with multiple scattered Remak bundles generally containing fewer than 30 axons each (Figures 4 and 5 and Supplemental Figure 6). Nearly all (>98%) Remak axons were enveloped by an SC membrane overlying each axonal membrane. Amyelination patches (large bundles of variable-caliber naked axons lying outside SCs) were completely absent. In contrast, the untreated *dy^2J^/dy^2J^* sciatic nerves contained large plaque-like, amyelinated patches. The ultrastructural appearance of these patches was that of unmyelinated single axons seen to lie immediately adjacent to each other, each possessing a single axonal membrane with no intervening SC cytoplasm or enveloping membrane. In addition, the Remak bundles of *dy^2J^/dy^2J^* mice contained a mixture of enveloped and naked axons, the latter often present in groups of 4 to as many as 80 axons (Figure 5B). Many of the bundles were much larger than those of WT mice (Figure 5C). The dystrophic Remak bundles were bounded by discontinuous BMs (Figure 4D).

The *dy^2J^/dy^2J^* sciatic nerves from AAV-SPc5-12-αLNNd–treated mice were nearly identical in appearance to the untreated *dy^2J^/dy^2J^* sciatic nerves with respect to the presence of amyelination patches and large Remak bundles containing many naked axons. In contrast, the *dy^2J^/dy^2J^* sciatic nerves examined from mice treated with high- and low-dose AAV-CBh-αLNNdΔG2′ were essentially indistinguishable from WT sciatic nerves (Figures 4 and 5). They were completely devoid of amyelination patches (at both the light and electron microscopic levels), and the Remak bundles were small with their axons nearly all enveloped as seen in WT Remak bundles. The extent of myelination was measured by calculating the g-ratios (axonal/total fiber diameters) for methylene blue– stained sciatic nerve sections (excluding amyelination patches), overall and as a function of axonal area (Figure 5, D–H). WT, *dy^2J^/dy^2J^*, and treated *dy^2J^/dy^2J^* myelinated axons exhibited similar g-ratios (Figure 5D), a finding in agreement with measurements comparing WT with Lmα2^–/–^ sciatic nerves (35). The distribution of g-ratios for different axonal areas (Figure 5, F–I) suggests that there is a small population of axons that are undermyelinated in *dy^2J^/dy^2J^* nerve not expressing αLNNdΔG2′.

### Skeletal muscle.

Forelimb and hind limb muscle sections from 15-week-old mice were stained with periodic acid–Schiff (PAS) and picrosirius red (PSR). PAS was preferred over H&E because it more clearly delineated the sarcolemmal BMs while PSR was used to detect collagens as a measure of fibrosis. The stained sections of proximal and distal forelimbs and hind limbs were surveyed to identify the muscles with substantial myofiber damage (rounded myofibers of variable size, interfiber cellularity and ECM, high density of central nuclei) and collagen deposition (Supplemental Figures 7–11). Prominently affected muscles in the untreated *dy^2J^/dy^2J^* mice included extensor carpi radialis longus and brachioradialis in the distal forelimb and plantaris, soleus, and gastrocnemius in the distal hind limb. The greatest benefit of AAV9-SPc5-12-αLNNd treatment was seen in proximal forelimb muscles, vastus muscles, and tibialis anterior and extensor digitorum longus, whereas other regions exhibited slight to modest improvement. In contrast, AAV9-CBh-αLNNdΔG2′ treatment of *dy^2J^*/*dy^2J^* mediated a more substantial overall improvement of multiple muscles.

**Figure 6:**
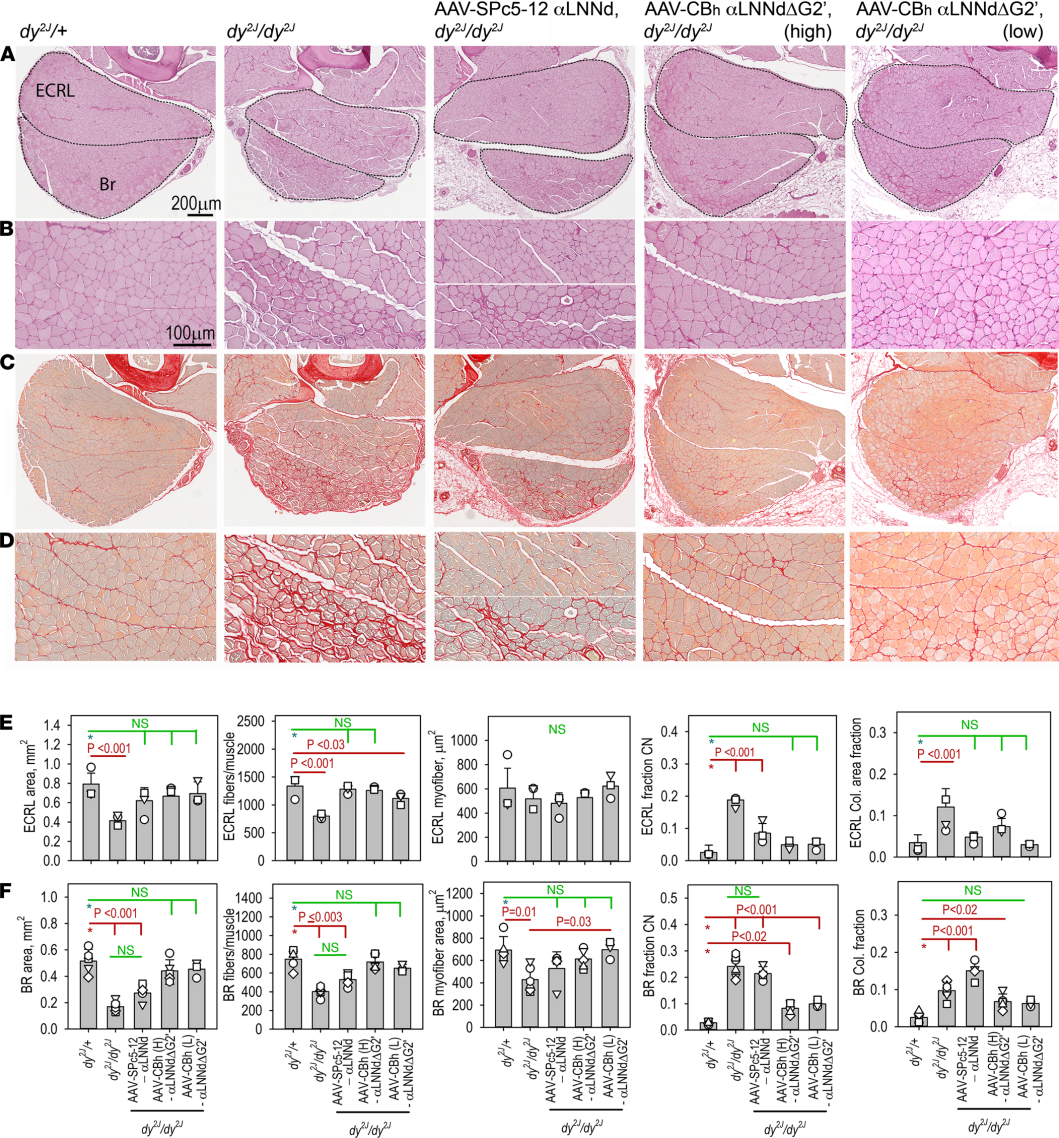
Histology of distal forelimb muscles. Extensor carpi radialis longus (ECRL) and brachioradialis (Br) are shown stained with PAS (**A** and **B**) and PSR (**C** and **D**) at 15 weeks (**A** and **C**, bar, 200 μm; **B** and **D**; bar, 100 μm). Bar graphs (av. ± SD with individual mouse values) for whole muscle areas, muscle myofiber number, myofiber areas, fraction of central nuclei within myofibers, and area of muscle staining for collagen with PSR are shown for ECRL (**E**) and Br (**F**). Statistical significance (**E** and **F**) was determined from the average and SD by 1-way ANOVA followed by Holm-Šidák test pairwise comparisons. Histological improvements of the dystrophy were seen after treatment with either virus preparation.

**Figure 7:**
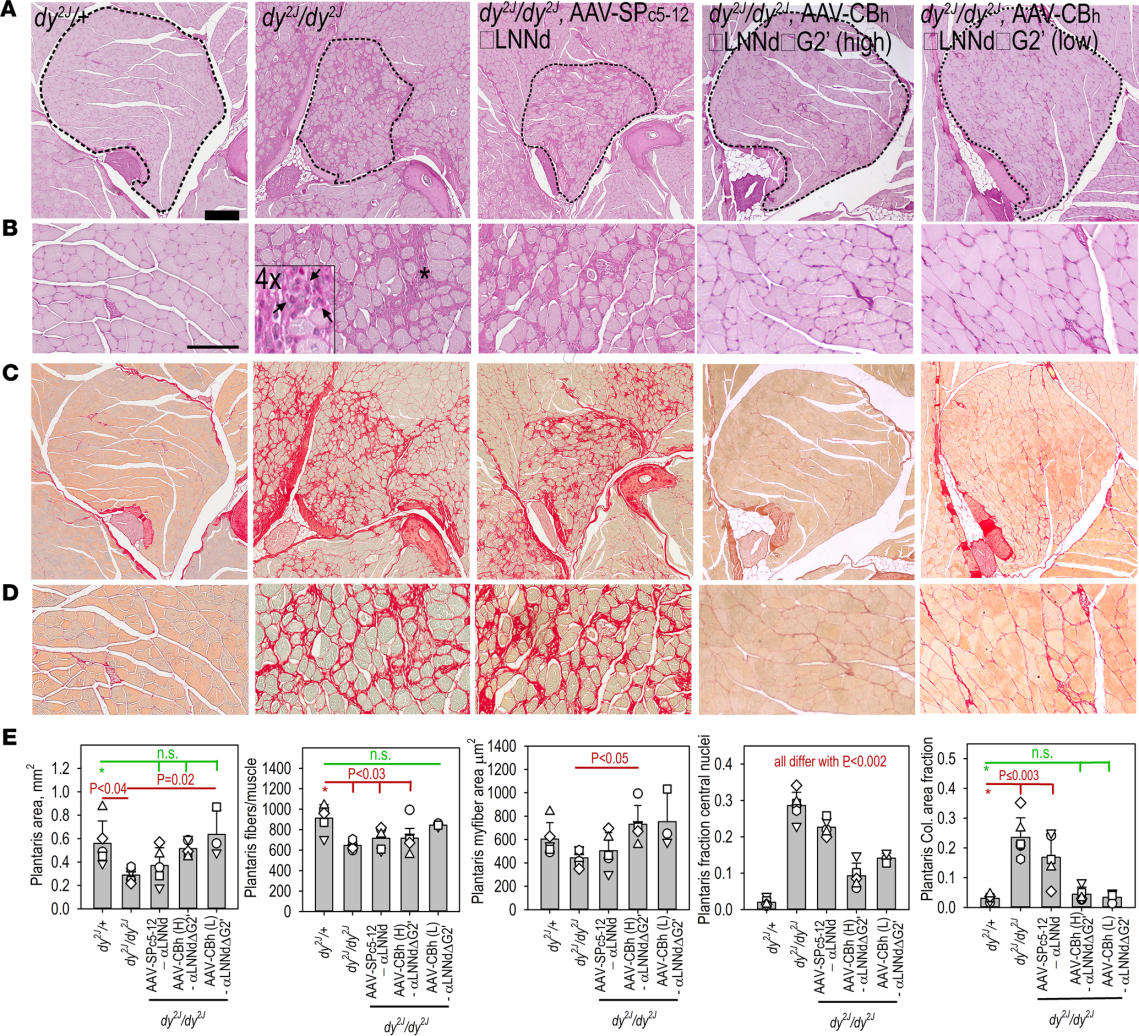
Histology of plantaris. Hind limb muscles from 15-week-old mice, *dy^2J^/+* (first column), *dy^2J/^dy^2J^* (second column), and *dy^2J^/dy^2J^*, treated with AAV-αLNNd (SPc5-12 promoter, third column), high-dose AAV-CBh-αLNNdΔG2′ (fourth column), and low-dose AAV-CBh-αLNNdΔG2′ (fifth column) and stained with either PAS (**A** and 3× detail, **B**) or PSR (**C** and 3× detail, **D**) (Bars, 200 μm, rows **A** and **C**; 100 μm, rows **B** and **D**). Asterisk in *dy^2J^/dy^2J^* PAS-stained muscle indicates location of commonly seen collection of small cells (arrows) as shown in 4× inset (12× compared with **A**). (**E**) Bar graph plots (average ± SD with individual mouse data points) of whole muscle areas, muscle myofiber number, myofiber areas, fraction of central nuclei within myofibers, and area of muscle staining for collagen with PSR are shown. Statistical significance (**E**) was determined from the average and SD by 1-way ANOVA followed by Holm-Šidák test pairwise comparisons.

Cross sections showed forelimb brachioradialis with extensor carpi radialis longus (Figure 6) and the hind limb muscle plantaris (Figure 7) were among the muscles more severely affected by dystrophic morphological changes in the untreated mouse. Sections of these muscles were evaluated by morphometry (Figures 6 and 7, and Supplemental Figure 12) to estimate muscle area (excluding nonmyofiber regions), myofiber number, single myofiber areas, fraction of myofibers with central nuclei (regenerating myofibers), collagen areas (fibrosis), and the distribution of myofiber cross-sectional areas. Plantaris exhibited particularly frequent small rounded myofibers, reduced numbers of myofibers, variable infiltration by small dark-staining cells (PAS) that correspond in appearance to chronic inflammatory cells, frequent myofiber central nuclei (20% to 30%), and peri-myofiber fibrosis in untreated *dy^2J^/dy^2J^* mice. Brachioradialis and plantaris showed limited histological improvement in response to treatment with AAV9-SPc5-12-αLNNd. In contrast, greater improvement was seen with AAV9-SPc5-12-αLNNd in extensor carpi radialis longus. In general, the muscles least improved following AAV9-SPc5-12-αLNNd treatment were those most severely affected in the untreated dystrophic mouse. On the other hand, AAV9-CBh-αLNNdΔG2′ treatment of dystrophic pups resulted in even greater improvements in the corresponding muscles, with muscles largely if incompletely devoid of dystrophic changes. Cross-sectional areas approached those of WT in brachioradialis and plantaris; the fraction of central nuclei, a measure of regeneration, was substantially reduced (to ~10%); and the extent of fibrosis was greatly reduced (to about half that of untreated in brachioradialis and equivalent to WT in plantaris).

### AAV linker protein expression and DNA in muscle, liver, and blood.

**Figure 8:**
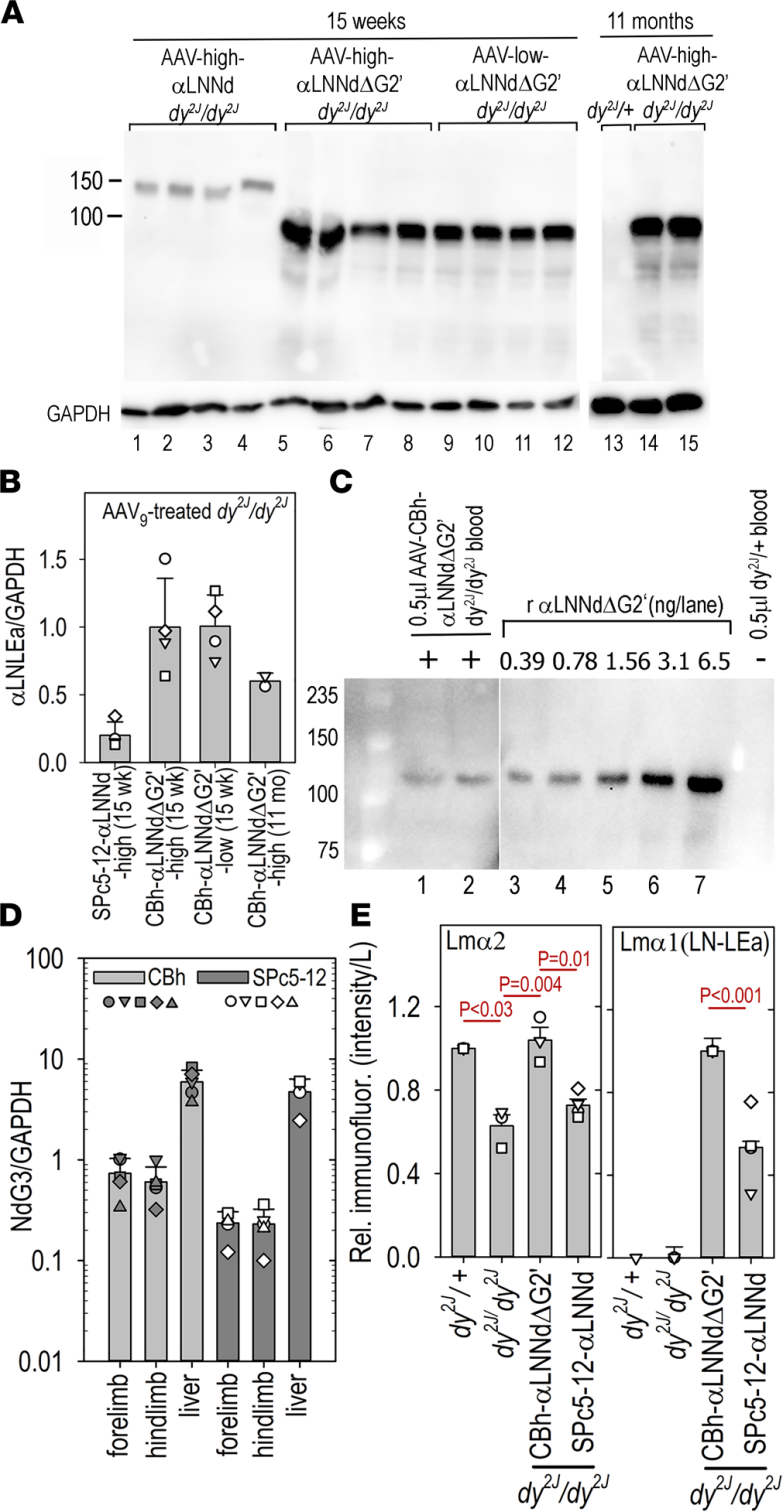
Linker protein levels in muscle. (**A**) Aliquots of muscle extracts from 15-week-old mice were subjected to SDS-PAGE (0.4 mg muscle, 7.5% acrylamide gel, reduced, and 0.04 mg muscle/lane, 10% gel for GAPDH). Following membrane transfer, linker protein was detected with rabbit anti–Lmα1LN-LEa. (**B**) Plot of relative average and SD values of linker protein/GAPDH bands with individual mouse data points are shown. Expression was still detected at 11 months. (**C**) Aliquots (0.5 μL) of blood from a *dy^2J^/dy^2J^* mouse treated with AAV-CBh-αLNNdΔG2′ and a *dy^2J^/+* mouse, along with different concentrations of recombinant αLNNdΔG2′ in WT blood, were compared in immunoblots following SDS-PAGE. The concentration of linker protein in blood is estimated to be 0.5–1 μg/mL. Linker protein was not detected in urine (not shown). (**D**) Quantitative PCR values (average and SD, 5 mice/condition) for 15-week proximal hind limb muscle (NdG3/GAPDH of 1 = 2 × 10^4^ vg/100 ng DNA). (**E**) Ratios of Lmα2 and α1 linker proteins (prox. hind limb, 9 weeks). Muscle from the indicated adult mice was immunostained with rat anti–N-terminal Lmα2 antibody (1:100) and rabbit anti-Lmα1 antibody (1:100) and detected with secondary Alexa 488 and 647 fluorescent antibodies. Images from 5–15 original magnification 10× fields/mouse (3–4 mice) were segmented with determination of summed intensities/BM length. Statistical significance (**B**, **D**, and **E**) was determined from the average and SD by 1-way ANOVA followed by Holm-Šidák test pairwise comparisons.

Immunoblots from muscle extracts were evaluated for linker protein from 15-week-old and 11-month-old treated mice (Figure 8, A–C). αLNNdΔG2′ was detected at higher levels in muscle compared with full-length αLNNd. Expression at 11 months appeared to be reduced but still above the level of expression upon treatment with AAV9-SPc5-12-αLNNd at 15 weeks. The linker αLNNdΔG2′ was also increased relative to αLNNd at 9 weeks by quantitation of muscle immunostained intensities in muscle BMs, with only the former linker appearing in peripheral nerve branches (Figure 8E). Quantitative PCR (qPCR) of extracts of muscle and liver to detect the nidogen-G3 domain of the 2 linker proteins (rendered unique by the codon-optimized DNA sequence) revealed that liver contained approximately 10-fold higher DNA compared with muscle and that linker DNA in muscle was 2- to 3-fold greater following use of the CBh promoter compared with the SPc5-12 promoter (Figure 8D). Given the ubiquitous nature of CBh-driven expression, it was thought that the linker protein might be present in blood. Blood from AAV9-CBh-αLNNdΔG2′–treated dystrophic adult mice was tested for the presence of linker protein by direct Western immunoblotting using α1LN-LEa–specific antibody (Figure 8C). αLNNdΔG2′ was detected as a full-size protein at an estimated concentration of 0.5–1 μg/mL. Since blood was found to contain αLNNdΔG2′ protein, this likely explains the greater intensity differences detected in whole muscle blots between αLNNdΔG2′ and αLNNd compared with immunostained intensity differences by comparing BMs. Linker protein was not detected in urine (blot not shown).

### Linker protein and laminin expression in brain and liver.

(a) Brain cortex of WT and *dy^2J^/dy^2J^* mice, untreated and treated with CBh-αLNNd-αLNNdΔG2′, was evaluated by immunofluorescence microscopy (Supplemental Figure 13). Laminin α2, readily detected in the brain cortex in capillaries, small vessels, and other cortical structures, was reduced in *dy^2J^/dy^2J^* and increased in *dy^2J^/dy^2J^* treated with AAV9-CBh-αLNNdΔG2′. Immunostaining of liver in AAV9-CBh-αLNNdΔG2′–treated dystrophic mice revealed linker protein expression in vessels and bile duct structures (Supplemental Figure 14).

## Discussion

LAMA2-RD is a muscular dystrophy and a neuropathy. The neuropathy is particularly evident in mouse models of the disease. An earlier study of transgenic muscle-specific expression of αLNNd linker protein in *dy^2J^/dy^2J^* mice revealed improvement of forelimb grip strength and histology but persistence of hind limb paresis and contractures (12). Based on such evidence, it was thought that peripheral nerve expression, in addition to that of muscle, would help optimize treatment of LAMA2-RDs and that if AAV somatic gene therapy was to be employed, the linker protein and corresponding DNA packaged in AAV would need to be reduced in size to accommodate a high-expression general promoter.

Laminin-binding chimeric laminin/nidogen linker proteins containing laminin LN and adjacent LEa domains were originally conceived as reagent tools to study the LN specificity of laminin polymerization (5, 14). Studies of BM assembly on cultured myotubes suggested that the αLNNd linker protein had potential for the treatment of laminin α2 polymerization deficiency (12, 13). In using αLNNd, α1LN activity replaced that of the inoperable α2LN by serving as a new functional α short arm to combine with existing βLN and γLN domains. However, while the size of αLNNd was ideal for polymerization studies and for transgenic expression in mice, it created a challenge in the choice of promoters because of the capsid size limitation. Importantly, it was anticipated that high αLNNd expression was needed to effect a meaningful change in BM structure because (a) laminin self-assembly is stoichiometric relative to other components and (b) the linker protein must compete against endogenous nidogens to achieve significant binding to the laminin γ1 subunit. If nonessential domains within αLNNd could be identified, they could be eliminated, allowing for the choice of larger universal promoters and stabilizing elements. The nidogen-derived G2 domain that mediates αLNNd binding to collagen IV and perlecan is the largest internal domain. Removal of the large nidogen-derived G2 domain along with flanking domains reduced the protein length to 1003 residues (DNA, 3.01 kb). Analysis of linker proteins lacking G2 revealed that it is not critical for cell surface BM assembly since collagen IV and perlecan could be recruited to laminin by nidogen-1 in the incubation mix. Since the αLNNdΔG2′, tethered to a nonpolymerizing laminin, cannot bind to collagen IV or perlecan, only endogenous nidogen can provide these important activities. This induces heterogeneity of the BM polymer architecture in which none of the polymerizing laminin can bind nidogen, with only nonpolymerizing laminin tethered to endogenous nidogen forming strong linkages with the collagen IV polymer.

Temporal vein injection of neonatal dystrophic pups with the 2 different modified viruses led to muscle-specific expression of αLNNd and multiorgan protein expression of αLNNdΔG2′ that included muscle, peripheral nerve, and vasculature of muscle, peripheral nerve, brain, and liver. qPCR revealed the presence of αLNNd and smaller αLNNdΔG2′ linker in muscle and liver. Muscle immunofluorescence intensity and extract levels were higher for αLNNdΔG2′ compared with αLNNd, even when AAV was delivered to mice at a lower dose. Western immunoblots of muscle extracts revealed higher overall αLNNdΔG2 protein levels compared with αLNNd relative to immunofluorescence differences, probably a consequence of liver and/or vascular endothelial expression in blood. However, it is unclear if the linker protein in blood is readily transported to muscle and SC BMs such that it can contribute to BM assembly.

Immunofluorescence of muscle revealed higher intensities and more evenly distributed expression with CBh-αLNNdΔG2′ compared with SPc5-12-αLNNd of both the α1 N-terminal and α2 laminin epitopes. The laminin α2 epitope was brighter in *dy^2J^/dy^2J^* mice treated with either linker compared with untreated. In contrast, a characteristic of the SPc5-12-αLNNd–treated dystrophic muscle was presence of scattered focal regions in the muscle cross sections in which expression of both epitopes was reduced. These regions showed many smaller and rounded myofibers and intervening matrix as seen in untreated dystrophic muscle. It may be that there was locally insufficient polymerizing laminin to support myofibers, leading to their degeneration and death. Since the AAV-delivered DNA remains episomal and is not capable of duplication, regenerating myofibers replacing the damaged ones would lack the ability to express the linker. This phenomenon was not detected following CBh delivery in which higher likely protective expression was achieved.

Distal forelimb and distal hind limb muscles were generally more severely affected, such as extensor carpi radialis, brachioradialis, plantaris, and gastrocnemius. Delivery of αLNNdΔG2′ resulted in amelioration to levels approaching those of WT in nearly all muscles examined (with the least benefit seen in brachioradialis and plantaris). While the degree of improvement to muscle strength may depend on the dose of AAV, the lower-dose delivery of αLNNdΔG2′ still substantially improved grip strength without observable reduction in the histological improvement of either muscle or peripheral nerve. Muscle-specific expression of the linker protein LNNd provided significant amelioration of forelimb muscle weakness and pathology as well but only more limited amelioration of hind limb. These improvements largely mirrored those seen with muscle-specific transgenic expression of LNNd in *dy^2J^/dy^2J^* mice (12).

While laminin α2 was barely detected in untreated *dy^2J^/dy^2J^* sciatic nerve and branches, intense expression of Lmα1 and Lmα2 was observed in peripheral nerve following CBh-αLNNdΔG2′ viral treatment. The absence of naked axons in the CBh-treated *dy^2J^/dy^2J^* sciatic nerves (both high and low doses), as well as the absence of characteristic hind limb weakness and contractures in the corresponding mice, was striking, providing evidence in a mouse model to support in vitro findings that laminin polymerization is required for radial axonal sorting and that it can be corrected with an appropriate laminin-binding linker protein.

Patients with LAMA2-RDs characteristically exhibit white matter brain changes by magnetic resonance imaging (MRI), with some exhibiting brain atrophy and neuronal migration defects, with symptoms often including seizures (6). While mechanisms underlying the MRI changes and seizures remain unclear, blood-brain barrier capillary leakage has been reported in LmC1/nestin-cre knockdown and *dy^3K^/dy^3K^* mice (36, 37). Future studies of dystrophic mice following linker protein expression in the brain will be needed to determine if the increased microvascular expression of linker protein and α2-laminins benefits brain function.

Overall, improvements of mouse activity, grip strength, and histology in disease-affected tissues were more extensive with the shortened version of LNNd using the CBh promoter, possibly aided by the WPRE enhancing element. In summary, we conclude that the difference in benefit between the 2 AAV constructs results from the higher muscle expression of a linker achieved with a general promoter, the added expression in peripheral nerve that prevented amyelination, and possibly increased laminin α2 expression in brain as well. The CBh promoter benefit was seen at both high- and low-dose treatment, with even the low dose outperforming high-dose muscle-specific delivery.

AAV-based treatment of laminin α2 deficiency to achieve a structural repair of BMs with laminin, laminin domains, or other BM components represents a new approach to therapy (1). In addition to expression of laminin-binding linker as described in this study, there exist strategies to activate a substitute laminin gene (10) and to express laminin domains that provide missing activities (11). For example, AAV directed CRISPR/Cas to activate laminin α1 as a compensatory subunit in *dy^2J^/dy^2J^* mice produced improvements in motor strength, muscle histology, and peripheral nerve (10). In that study, 2 AAV viruses were used in neonatal pups. The approach of full subunit substitution was based on observations that transgenic expression of the laminin α1 subunit substantially ameliorated the dystrophy of the *dy^3K^/dy^3K^* mouse (38, 39). AAV9 was employed as the serotype of choice because of its ability to target muscle and nerve in the current study. As seen with many AAV serotypes, high expression was seen in liver with a potential for untoward hepatic consequences in patients. Recently, the AAV capsid has been engineered to more efficiently selectively target muscle with greatly reduced liver expression (40). Continued engineering of AAV may produce capsids that also target the neuronal tissues affected in LAMA2-RDs, a desired characteristic to optimally treat the neuromuscular defects of LAMA2-RDs.

The great majority of LAMA2-RDs result from a total or near-total reduction of expression of the laminin α2 subunit, characterized by greater severity of symptoms (1). In *dy^3K^/dy^3K^* and *dy^W^/dy^W^* mice, the absent or near-absent laminin α2 subunit is compensated by overexpression of the laminin α4 subunit that lacks αLN and receptor-binding activity characteristic of α2-laminins (13, 41). In addition to loss of polymerization, α4-laminins bind poorly to α-dystroglycan (13). This receptor attachment, linking the BM to the cytoskeleton, can be restored in the compensatory laminin by binding of the coiled-coil domain of laminins to the protein miniagrin (mag) (41). Mag, like αLNNd, was found to separately improve muscle histology and function when delivered by a transgenic route and by AAV somatic gene therapy (13, 41–43). Combined muscle-specific transgenic expression of αLNNd and mag resulted in a considerably greater improvement in muscle histology and function in the *dy^W^/dy^W^* mouse compared with either component alone (13). Based on such findings, there is an expectation that a combined treatment of the severely affected dystrophic mice that enables both polymerization and receptor anchorage will enable a more effective amelioration of the most common form of the disease.

## Methods

### DNA constructs.

Expression vector for the mouse αLNNd is as previously described (14, 27). Removal of the G2 nidogen-1 domain in αLNNd pcDNA3.1 zeo was accomplished with overlapping PCR. In the first round of PCR, a 1.2 kb-5′ (1F 5′-ctgggtcactgtcaccctgg-3′ and 2R 5′-atggattctgaagacagacaccagagacac-3′) and 1.8 kb-3′ (2F 5′-ctggtgtctgtcttcagaatccatgctac-3′ and 1R 5′-gaaggcacagtcgaggctgatcag-3′) product was generated on either side of the G2 nidogen-1 domain of αLNNd. They were sewn together with a second round of PCR (1F and 1R) into a 3 kb product, which was then digested with EcoRI to 2.4 kb and ligated into the 5.85 kb EcoRI αLNNd pcDNA 3.1 zeo vector (generating an 8.25 kb αLNNd ∆G2^c^ pcDNA3.1 zeo plasmid). A further 2 EGF (270 bp) deletion of αLNNd ∆G2^c^ was performed with overlapping PCR primers (Bam 1F 5′-cggcagcctgaatgaggatccatgcataga-3′ and 2R 5′-cacagtagttgatgggacagacacc-3′) and 3′ (2F 5′-gtctctggtgtctgtcccatcaacta-3′) and Sse 1R 5′-gaggcacaaacatcccctgcagggtgggcc-3′ to generate 160 bp and 357 bp products, respectively. After a second round of PCR, a 485 bp BamHI-SbfI digested insert was ligated into a likewise digested αLNNd ∆G2^c^ pcDNA3.1 zeo vector (7.5 kb), generating αLNNd ∆G2′^c^. To remove the N-terminal Myc tag on the αLNNd ∆G2′^c^ open reading frame (ORF), a 1.5 kb BamHI insert was moved from the αLNNd mck-pA construct to the MCS-AAV vector (4.6 kb Cell Biolabs, VPK-410-DJ), generating a 6.1 kb AAV-5′ αLNNd no tag-10 plasmid. The LF fragment was removed with 1F 5′-caatggaaagtggccaggtcagt-3′ and 2R 5′-cacagtagttgatgggacagacacc-3′ as well as 2F 5′-cgagggctgctccatcaactactgtg-3′ and 1R 5′-ctgatcagcctcgactgtgccttc-3′, generating 296 bp and 1542 bp products, respectively. They were sewn together with the same 1F and 1R primers, digested with XagI-XhoI, and inserted into αLNNd ∆G2^′c^ pcDNA3.1 zeo for plasmid αLNNd ∆G2′ pcDNA3.1 zeo. A nontagged αLNNd mck-pA was generated after replacing the N-terminal myc-tag from αLNNd BS mck+polyA (12) with a similar BamHI insert from mα1 pCis (27). The αLNNd ∆G2^′c^ pcDNA3.1 zeo plasmid was digested with FseI and XhoI to generate a 2.8 kb insert, which was ligated into the similarly digested AAV-5′ αLNNd no tag-10 vector (4.9 kb). The final vector size was 7.7 kb with an ORF (αLNNdΔG2′) of 3009 bp.

### AAV virus and qPCR analysis.

Viral constructs αLNNd and αLNNdΔG2′ were codon optimized and synthesized by VectorBuilder with appropriate inverted terminal repeat, promoter, Kozac, poly A tail, and WPRE. All AAV9 preparations were produced by Vigene Bio. Genomic DNA was extracted with Qiagen DNeasy Blood & Tissue Kit (catalog 69504). Virus titer was determined with qPCR (AAVpro titration kit, Takara Bio; QuantiFast SYBR Green PCR Kit, Qiagen catalog 204056). All qPCR primers for αLN virus constructs were generated by Integrated DNA Technologies (primer 1 5′tgttcatcgctgcagtagtt3′ primer 2 5′atcagcaaggagatggacac3′). Viral titers and genome copy number were compared to the αLNNdΔG2′ genome standard curve and GAPDH (Qiagen QuantiTech catalog 249900).

### Recombinant and native laminins and other proteins.

(a) HEK293 cell lines (ATCC) stably expressing recombinant laminin 111 heterotrimers (WT and deletion-modified), αLNNd, and αLNNdΔG2′ were purified by FLAG or HA affinity chromatography as described in detail (28). (b) Recombinant mouse nidogen-1 was purified from conditioned medium by HisPur-cobalt chelating chromatography (Thermo Fisher Scientific, catalog 89965) and detected with a rabbit polyclonal antibody as described (14) or with anti-nidogen (Chemicon catalog MAB1946). (c) Type IV collagen was extracted from lathyritic mouse Engelbreth-Holm-Swarm (EHS) tumor and purified as described (44). (d) Mouse recombinant perlecan was purified from conditioned medium of transfected cells by diethylaminoethyl Sephacel (Sigma catalog I6505) and HisPur-cobalt affinity chromatography (Thermo Fisher Scientific catalog 89964) as described (45).

### Protein determinations.

Molar laminin concentrations were determined by densitometry of Coomassie blue–stained acrylamide gels in comparison to an EHS-laminin (710 kDa protein mass) standard (46) corrected for changes in calculated mass (LmΔαLN-L4b, 558 kDa), Lm411 (513 kDa), Lm511 (784 kDa), and Lm211 (718 kDa) as previously described (14, 27). Absorbance at 280 nm was used to measure the concentration of αLNNd (157 kDa) and αLNNdΔG2′ (112 kDa).

### Culturing, immunostaining, and analysis of BM assembly in C2C12 myotubes and rat SCs.

(a) Mouse C2C12 myoblasts (ATCC CRL-1772) were maintained and differentiated into myotubes based on a method as described (47). Myoblasts were maintained in DMEM, 10% fetal calf serum, and penicillin-streptomycin. Cells between passages 6 and 7 were plated onto 24-well dishes at 250,000 cells per well and incubated at 37°C overnight. The following day, the media were changed to DMEM, 9% horse serum/1% fetal bovine serum (Gibco), and 1% penicillin-streptomycin, and cells were allowed to fuse for 4 to 5 days at 37°C. (b) After 1-hour incubation with protein samples, myotube cultures were washed 3 times with phosphate-buffered saline (PBS) followed by fixation in 3.5% paraformaldehyde in PBS for 30 minutes at room temperature (RT). Cultures were blocked overnight at 4°C with 5% goat serum and 0.5% BSA in PBS. Myotube lawns were stained with 1 μg/mL primary polyclonal and 1:100 monoclonal antibodies specific for laminin subunits, laminin domains, nidogen-1, and collagen IV as previously described (12). (c) Laminin and BM assembly were evaluated on rat SCs adherent to plastic as previously described (5). (d) Detection of bound primary antibodies was accomplished with Alexa Fluor 488 goat anti-rabbit (catalog A-11034), Alexa Fluor 647 anti-chicken (catalog A-21449), and Alexa Fluor 647 anti-mouse (catalog A-21235) IgG secondary antibodies (Molecular Probes) at 1:100, with nuclear counterstaining with DAPI (48). Myofiber cultures were viewed by indirect immunofluorescence using an inverted microscope (model IX70; Olympus) fitted with an IXFLA fluorescence attachment and a charge-coupled device camera controlled by IP Lab 3.7 (Scanalytics). (f) Digital images were recorded (5–12 fields, each 1300 × 1030 pixels) using a 10× microscope objective with the same exposure time for a given primary and secondary antibody set when comparisons were to be made. Fluorescence levels were estimated from the digital images with ImageJ software (NIH) with calculations performed in Microsoft Excel. Overall background was calculated as the background/pixel multiplied by the total area of myotube field occupying the field and subtracted from the sample fluorescence. Data were expressed as the mean ± standard deviation of normalized summed intensities in SigmaPlot 12.5 (Systat).

### Mice.

(a) *dy^2J^*/+ mice (C57BL/6J background) were purchased from Jackson Laboratories and maintained for breeding of dystrophic mice. (b) For genotyping, genomic DNA was purified from mouse tail clippings obtained at the time of weaning in 0.1 M NaOH buffer and boiled at 100°C in PCR tubes for 15 minutes. Samples were cooled, vortexed, and diluted into 40 mM Tris buffer, pH 8.0. Genotyping PCRs were done with 1 μL of genomic DNA per 20 μL reaction according to the Jumpstart Taq (MilliporeSigma P2893) instructions. PCRs were performed for *dy^2J^* mice with primers at final concentrations of 5 pmol/μL using *dy^2J^* forward 5′-tcctgctgtcctgaatcttg-3′ and *dy^2J^* reverse 5′-cattctgtgccagggagtc-3′ to generate a 300 bp product under the following thermocycler conditions: 94°C for 5 minutes; and 35 cycles of 94°C for 5 seconds; 52°C for 30 seconds; 72°C for 1 minute; ending with 72°C for 3 minutes. Following amplification, the PCR products were digested for 2 hours at 37°C with Thermo Fisher Scientific Fast Digest NdeI enzyme to yield a 300 bp, 200 bp + 100 bp, or 100 bp banding pattern when run on a 2% agarose gel to indicate WT, *dy^2J^*/+, or *dy^2J^*/*dy^2J^* genotypes, respectively (16).

### AAV9 vector delivery of DNA to mice.

Postnatal day 1 *dy^2J^/dy^2J^* pups (~1.4 g each) were injected via the temporal vein with either AAV9-SPc5-12-αLNNd at 3.8 × 10^11^ vg/g, AAV9-CBh-αLNNdΔG2′ at 4.2 × 10^11^ vg/g (high-dose group), or 1.1 × 10^11^ vg/g (low-dose group). WT (+/+ and *dy^2J^/*+), dystrophic (*dy^2J^/dy^2J^*), and AAV-treated dystrophic (AAV9-injected *dy^2J^/dy^2J^*) mice were weaned at 3 weeks of age and evaluated for specific grip strength of forelimbs, hind limbs, and all limbs between 3 and 15 weeks of age as well as lower limb activity and ambulation at different ages by inspection and video recording.

### Tissue harvesting and mouse tissue histology.

Forelimbs and hind limbs were dissected from euthanized mice at 9, 11, or 15 weeks of age. (a) For paraffin embedding, forelimbs and hind limbs were fixed in 10% buffered formalin overnight (MilliporeSigma SF93-4) after removal of skin. Specimens were incubated overnight at RT with an acidic decalcification solution (Fisher Cal-Ex CS510-1D), washed in running tap water for several hours, refixed in 10% formalin overnight, rinsed, cross-sectioned, and maintained in 70% ethanol for tissue processing. Tissues were embedded, sectioned at 5 μm, and stained with periodic acid H&E, PAS, and PSR at Rutgers Pathology Services. Panoramic images of proximal and distal forelimbs and hind limbs were generated by scanning PAS- and PSR-stained slides with a Leica Slide Scanner analyzed with Aperio ImageScope software at 3.96 pixels/μm. Selected regions-of-interest images were saved for image analysis and figure preparation. (b) Collagen level increases over baseline WT were estimated by determination of the area of red staining of selected digitally trimmed muscle cross sections from different mice stained in parallel (49). A segmentation range was determined from a PSR-stained *dy^2J^*/*dy^2J^* image and applied to all images evaluated in the set using ImageJ software. That segmentation value (sum value in pixels) was divided by the area (in pixels) of the muscle group to achieve the fibrotic area fraction. (c) For frozen sections, unfixed limbs were cross-sectioned, mounted, oriented in OCT, and rapidly frozen in liquid nitrogen. (c) A proximal segment of sciatic nerve was dissected from mice immediately after euthanasia and fixed in glutaraldehyde.

### Antibodies.

Generally, rabbit and chicken polyclonal antisera were affinity purified on respective ligands by affinity chromatography and cross-absorption. Columns of 3–4 mL of Sepharose-4B coupled to ligands were prepared by cyanogen bromide at 1–2 mg/mL ligand/mL beads. Loaded samples were eluted with 0.1 M acetic acid, with pH adjusted to neutrality, and dialyzed into PBS. For cross-absorption, the antibody was passed through the column in PBS with retention of the unbound fraction. Cross-absorption was repeated until adequate specificity could be demonstrated by ELISA and Western immunoblot determinations (protein in muscle lysates and recombinant Lm111, Lm211, Lm411, and Lm511). Polyclonal antibody specific for Lmα1LN-LEa domains was prepared by immunizing rabbits with recombinant Lmα1LN-LEa (3) followed by affinity purification on a laminin-αLN-LEa column and cross-absorbed against laminin 211, laminin 511, and β1LN-LEa (1). In muscle and peripheral nerve sections, the antibody immunostained only the BMs of mice in which αLN linker protein (αLNNd, αLNNdΔG2′) was expressed. Antibodies specific for laminin subunits α2 (mAb specific for α2L4b), α4, α5, nidogen-1, and collagen IV were used as described (12).

### Tissue preparation, antibody staining, and microscopy.

(a) Hind limb muscles were embedded in OCT (Tissue-Tek), flash-frozen in liquid nitrogen, and stored at –80°C. For sectioning, frozen blocks were first allowed to equilibrate to –20°C overnight. Five-micron-thick sections were cut with a cryostat (Leica CM 1850) at –20°C and adhered to positively charged slides (Thermo Fisher Scientific). Sections were then washed for 5 minutes in TBS-50 followed by fixation in 3.2 % paraformaldehyde in PBS for 15 minutes at room temperature. Slides were washed in PBS and blocked in 5% goat serum overnight at 4°C. Primary antibodies were added the following day for 1–3 hours at RT and washed 3 times in PBS for 10 minutes. Secondary antibodies conjugated with fluorescent probes were added for 1 hour at RT, followed by 30 minutes of PBS washes changed every 10 minutes. Slides were mounted with coverslips in 6% 1,4-diazabicyclo[2.2.2]octane in glycerol. Detection of bound primary antibodies in fixed frozen sections was accomplished with Alexa Fluor 488 and 647 goat anti-rabbit, chicken, and mouse IgG secondary antibodies (Molecular Probes) at 1:100. Tissue sections were stained simultaneously and antibodies titered to ensure linear detection of BM components. Regions of muscle were matched between genotypes, and the same exposure times and normalizations were applied to all images being compared. (b) In order to estimate and compare the immunofluorescence intensities for different laminin subunits, multiple near-adjacent original magnification 20× fields of cross sections from muscle were recorded and subjected to image segmentation analysis in ImageJ. A mean threshold signal value was determined to correspond to the BM zone of the image followed by collection of area and intensity density (sum) values. A mean background/pixel value for the non-BM zones was also determined by the same method, multiplied by the number of BM pixels, and subtracted from the BM summed values. To normalize each image, summed BM intensity was divided by BM length. Images were first processed with the ImageJ Find Edges algorithm followed by quantitation of the mean threshold signal values. An average and standard deviation was then calculated. (c) Freshly dissected sciatic nerve segments were fixed in 0.5% glutaraldehyde and 0.2% tannic acid in PBS and processed for electron microscopy as described (50). Semithin (1 μm) sections (~90 nm) were stained with 1% methylene blue in 1% sodium borate and imaged by scanning the slides with a Leica Slide Scanner as described for muscle above.

### Morphometry.

(a) Forelimb and hind limb skeletal muscle: PAS and PSR muscle cross-sectional areas were recorded from bright-field images. Muscle fibers were separated from the surrounding extracellular material including fibrosis as well as nerve and vasculature with pixel erasure in Adobe Photoshop. The isolated muscle fibers were then analyzed for muscle areas following segmentation in ImageJ. Myofiber numbers and central nuclei were determined by direct count of PAS-stained images in ImageJ. Average myofiber areas were estimated from the overall area divided by the number of myofibers. For evaluation of fibrosis, the PSR-stained extracellular regions were identified by segmentation analysis in ImageJ. The segmentation values, once determined, were similarly applied to all images within a group for analysis. (b) Axonal area and g-ratios of the myelin sheath were measured from methylene blue cross section images (original magnification 40×) with the software program “MyelTracer” as described (51). (c) The number and cross-sectional areas of amyelinated patches were measured in ImageJ. (d) Electron microscopic images of sciatic nerve: Multiple images of myelinated axons, Remak bundles, and amyelinated plaques (when present) were recorded at 3000× and higher magnification from the sciatic nerves of the different mice. The number of axons per Remak bundle and the fraction of these that were naked (nonenveloped) within each Remak bundle identified in over 10 Remak bundles/mouse/condition were recorded. Axons were scored as naked if the outer SC–derived membrane covered less than 50% of the axon.

### Muscle extraction and immunoblotting.

To make whole muscle extraction, upper leg muscle was pulverized frozen, then solubilized in a Polytron at 50 mg/mL in RIPA buffer. A total of 0.04 to 0.4 mg muscle/lane was loaded onto 7.5%–10% SDS-PAGE (for αLN or GAPDH respectively) and evaluated by Western blotting. Specifically, samples were boiled in Laemmli solubilizing buffer containing β-mercaptoethanol, loaded into gel lanes, and electrophoresed (1 hour, 130 volts). Gel-embedded proteins were transferred onto PVDF membranes in cold transfer buffer (25 mM Tris base, 190 mM glycine, 0.1% SDS, 20% methanol, pH 8.3) at 100 volts for 1 hour. Membranes were blocked overnight in 5% milk protein (50 mM Tris, 150 mM NaCl, 0.2% Tween 20). Primary antibody against either laminin-αLN-LEa (1 mg/mL) or GAPDH (MilliporeSigma G9295; 1:35,000 HRP primary Ab) was added in Western antibody buffer (50 mM Tris, 150 mM NaCl, 1% BSA, 0.2% Tween 20) for 3 hours at RT followed by three 5-minute washes with Western Wash Buffer (50 mM Tris, 150 mM NaCl, 0.2% Tween 20). Secondary antibody (goat anti-rabbit HRP conjugated, Thermo Fisher Scientific catalog 31460) was added at 1:3000 in Western antibody buffer for 1 hour at RT with gentle agitation. Membranes were washed 3 times (10 minutes each) and exposed simultaneously with equal volumes of West-Femto Kit components (Enhancer Solution + Peroxide Buffer) on the Bio-Rad gel-dock system and evaluated with Quantity One software.

### Grip strength measurements and video recordings.

(a) Grip strengths were measured with a mouse Grip-Strength Meter (Columbus Instruments) according to the manufacturer’s instruction (User Manual: 0167-007). Forelimb measurements were performed with the wire triangle attachment (pulling the mouse rearward by the tail) while hind limb and all limb measurements were performed with the angled grid attachment (pulling the mouse forward by the scruff of the neck with/without lifting the mouse by the tail to engage hind limbs or all limbs). The recommended 5 consecutive measurements/mouse protocol was increased to 10 with averaging of the 6 (rather than 3) highest values (12). Data were expressed as grams peak force divided by the mouse weight. (b) Videos of mouse movements were recorded with an iPhone12 with reduction of file sizes using VEED.IO and Wondershare Filmora editors.

### Statistics.

Averages and standard deviations were calculated from measured values obtained from 3 or more images and morphometric measurements of perimeters and areas with the statistical package in SigmaPlot 12.5 or Microsoft Excel. Averages and standard errors of the mean were determined from the means of consecutive sets of determinations (e.g., grip strength) from different mice and from the means of myofiber cross-sectional areas from different mice. Three or more conditions were compared by 1-way ANOVA followed by Holm-Šidák pairwise analysis in SigmaPlot while 2 conditions were evaluated by 2-tailed *t* test in Microsoft Excel. A difference was considered significant for *P* values ≤ 0.05 and trending toward significance for *P* values >0.05 to ≤0.10.

### Study approval.

The mouse protocol (9999-00384) for the study was approved by the Rutgers University — Robert Wood Johnson Medical School IACUC. The biosafety protocol (IBC 13-574) for the study was approved by the Institutional Biosafety Committee of Rutgers University.

## Author contributions

KKM and PDY developed the concept, planned the experiments, and wrote the manuscript. KKM prepared constructs and recombinant proteins, conducted in vitro analysis, cell culturing, immunostaining, microscopy, image analyses, qPCR, tissue extractions, and immunoblots. PDY oversaw the project; conducted morphometry, mouse husbandry, and behavioral and grip strength analyses; and prepared video recordings.

## Supplementary Material

Supplemental data

Supplemental video 1

Supplemental video 2

Supplemental video 3

## Figures and Tables

**Table 1:**
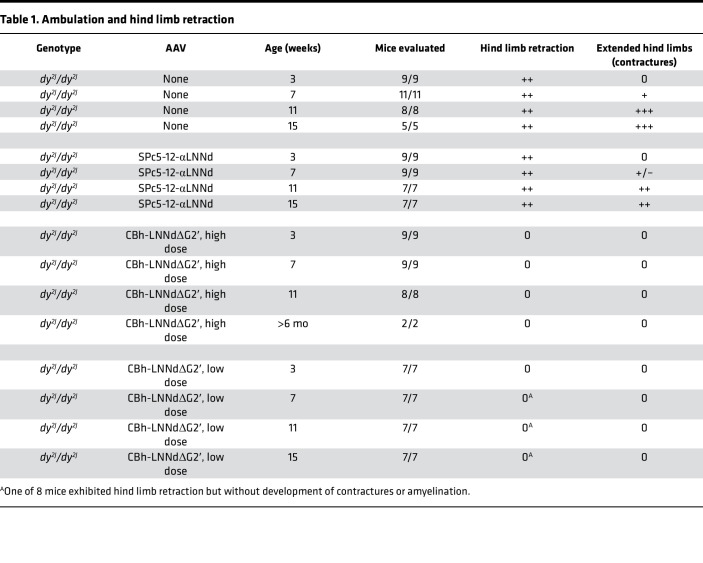
Ambulation and hind limb retraction

## Version 1 05/31/2022

In-Press Preview

## Version 2 07/08/2022

Electronic publication
