# Supplementary material for: Amelioration of muscle and nerve pathology of Lama2-related dystrophy by AAV9-laminin-αLN linker protein
Source: JCI Insight. 2022 Jul 8;7(13):e158397. doi: 10.1172/jci.insight.158397 (PMC9310540; doi:10.1172/jci.insight.158397)
Supplement: Supplemental data [file jciinsight-7-158397-s019.pdf]

Supplemental Figures and Tables

for

Amelioration of Muscle and Nerve Pathology of Lama2-related dystrophy by AAV9-Laminin-αLN-Linker Protein

Karen K. McKee and Peter D. Yurchenco

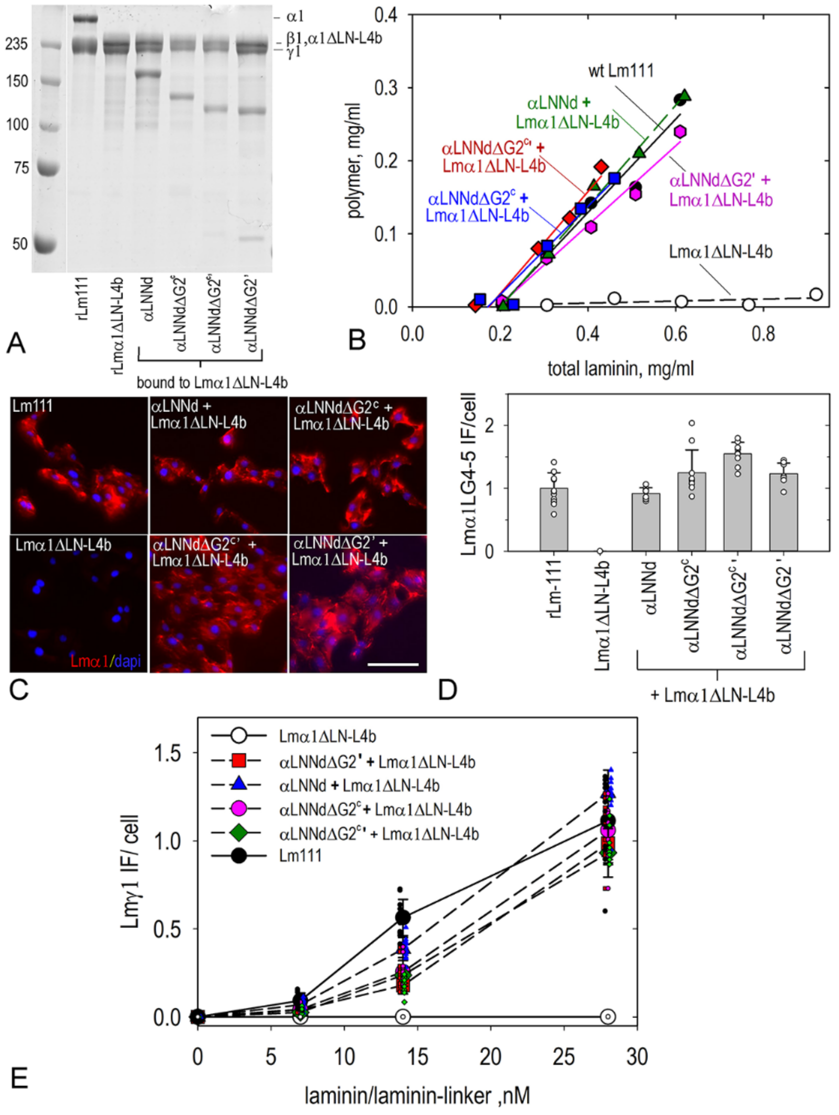

Supplemental Fig. 1. *Comparison of reduced-size Lmα1LN linker proteins*. **A**. The indicated proteins were coupled to Flag-tagged non-polymerizing recombinant laminin (Lmα1ΔLN-L4b), purified by affinity chromatography, and analyzed by SDS-PAGE under reducing conditions (Coomassie blue stain). **B**. Isolated WT Lm-111, Lmα1ΔLn-L4b and complexes of Lmα1ΔLn-L4b with linker proteins were evaluated in a polymerization assay. Following incubation, the solutions were centrifuged to separate polymer from the incubation mixes. All complexes polymerized similar to WT Lm-111. **C**. WT Lm-111 (rLm-111) and Lmα1ΔLn-LEa complexed to the indicated linker proteins (28 nM) were added to the conditioned medium of Schwann cells (SCs). After 1 hr, the cells were washed, fixed and stained with antibody to detect the laminin γ1 subunit (Bar, 100 μm). All protein complexes accumulated on cells similar to full-length αLNNd. **D**. Average +/- s.d. of different fields are plotted (n=8-12 10x fields). **E**. The different linker proteins were coupled to non-polymerizing Lmα1ΔLn-LEa at the indicated concentrations and added to the conditioned media of SCs. The cells were washed, fixed and stained with antibody to detect laminin-γ1 after 1 hour. Intensities are expressed as the average +/- s.d. (n=10-11 10x fields). Statistical significance (panels D, E) was determined from the average and s.d. by 1-way ANOVA followed by Holm-Sidak test pairwise comparisons. Superimposed single field determinations shown in D and E. The concentration-dependent accumulation of laminin was similar to that of fully intact Lm111 for all of the linker-modified conditions.

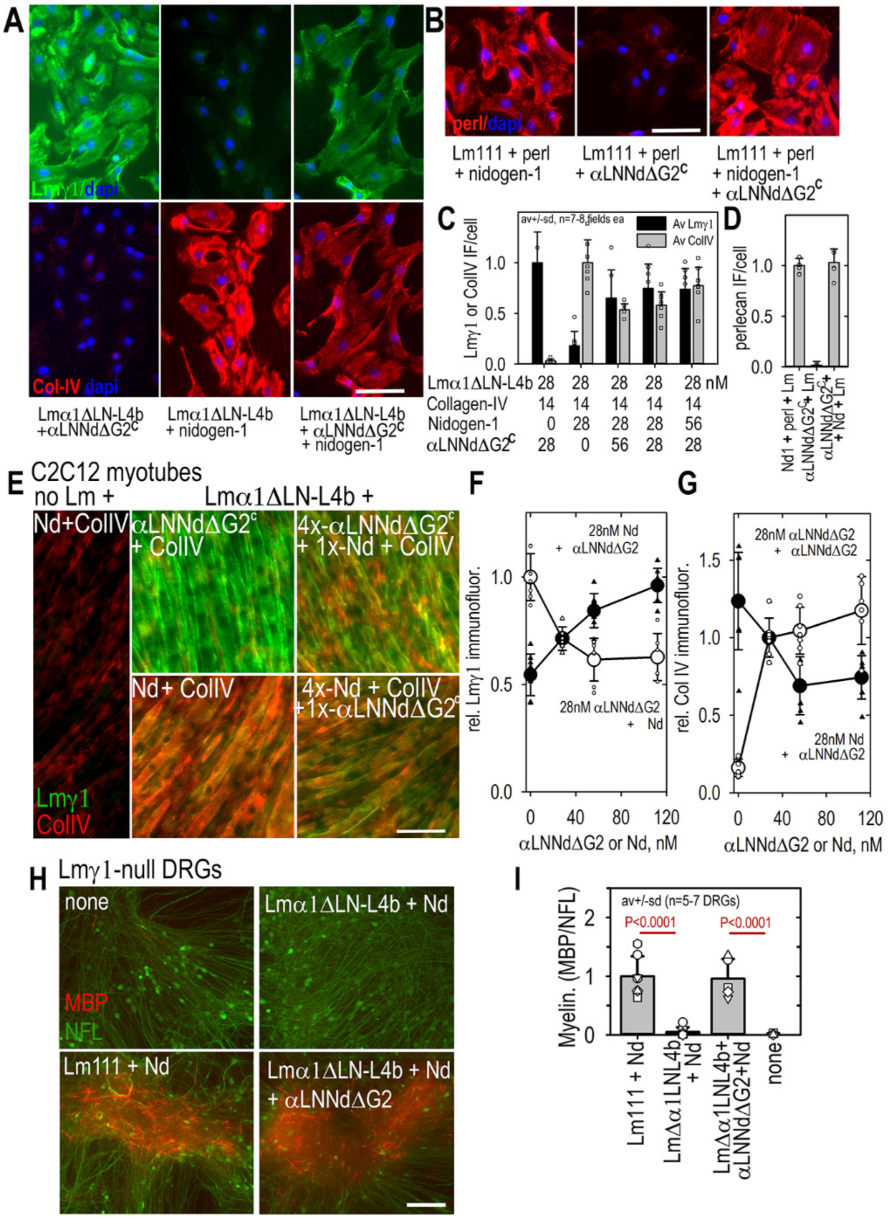

Supplemental Fig. 2. *Role of the nidogen G2 domain and its absence in a linker protein*. The DNA coding for the G2 collagen IV- and perlecan-binding domain of αLNNd was deleted in expression constructs. Recombinant protein (*αLNNdΔG2*^{C}) was purified, coupled to non-polymerizing Lmα1ΔLN-L4b, and evaluated for surface accumulation (assembly) on SCs and C2C12 myotubes. **A-D**. The linker protein, attached to Lmα1ΔLn-L4b (a non-polymerizing laminin lacking the α1 short arm), enabled laminin accumulation on SCs; however, the laminin was unable to recruit collagen-IV or perlecan on SCs unless also co-mixed with nidogen-1. Bar plots of intensities are shown in D and C (average ± s.d., n=6-8 10x fields per condition). **E-G**. A similar G2-dependency was observed on cultured myotubes. Plots of relative intensity are shown in panels F and G (average ± s.d., n=7-8 10x fields/condition). A competition between maximal laminin assembly and collagen-assembly, seen on both SCs and myotubes, depended on the ratio of linker protein to nidogen. (**H, I**) Myelination in Lmγ1-null dorsal root ganglia: DRGs excised from a pregnant E13.5 Lmγ1$^{fl/fl}$ mouse were grown in culture for 3 days and treated with Cre-adenovirus to inactivate the endogenous LamC1 gene. The cultures were then treated with either 14 nM Lm-111 + nidogen-1, Lmα1ΔLN-L4b + nidogen-1, or Lmα1ΔLN-L4b + nidogen-1 + αLNNdΔG2^{c} + ascorbate myelination medium. Bar plot shown in I (average ± s.d., n=5-7 10x fields). The linker protein enabled comparable myelination to that of WT Lm111. Myelin basic protein (MBP, red) and neurofilament (NFL, green) immunostaining were used to detect myelination and axons respectively. Length bars (A, B, E, 100 μm; H, 200 μm). Bar graph of intensities shown in I (average ± s.d.). Superimposed single field (C,D,F,G) and DRG values (I) shown. Statistical significance (panels C,D,F,G,I) was determined from the average and s.d. by 1-way ANOVA followed by Holm-Sidak test pairwise comparisons.

Lmα1ΔLn-L4b, only when coupled with linker protein, produced a similar degree of myelination compared to WT Lm111.

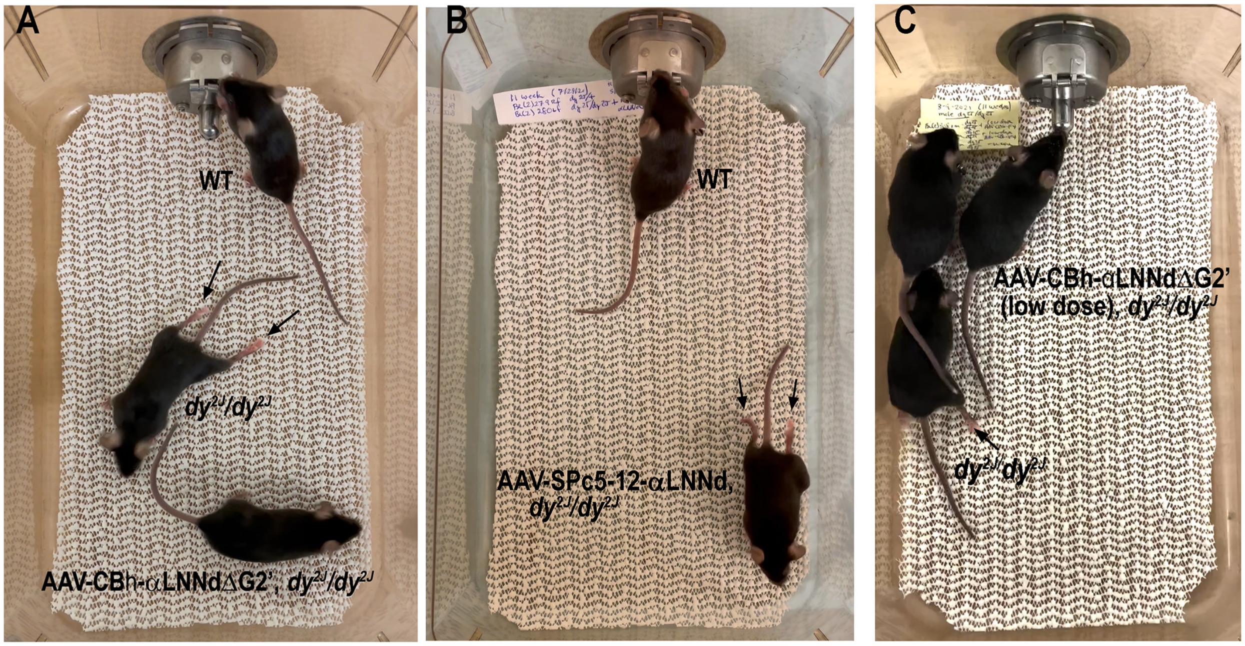

Supplemental Figure 3 and Video Recordings. *Mouse mobility.* **Panel A**: Representative WT (dy^{2J}/+), dy^{2J}/dy^{2J} and dy^{2J}/dy^{2J} mice treated with AAV$_9$-CBh-αLNNdΔG2'-high dose are shown (WT, no earring; dy^{2J}/dy^{2J}, right earing; AAV$_9$-treated dy^{2J}/dy^{2J}, left earing) at 11 weeks. The WT and AAV$_9$-treated dy^{2J}/dy^{2J} mice were indistinguishable with respect to general mobility and hindlimb function through 15 weeks of age. The untreated dy^{2J}/dy^{2J} mice developed progressive hindlimb paresis and extension contractures (arrows) that became permanent over time and that slowed ambulation. <u>See corresponding supplemental video "SupVideo1-hiCBh11wk.mp4</u>. **Panel B**: Comparison of a WT (dy^{2J}/+; right earing) with a dy^{2J}/dy^{2J} mouse treated with AAV$_9$-SPc5-12 αLNNd (left earing) at 11 weeks. The treated mouse exhibited developing hindlimb contractures (arrow) and gait was reduced. <u>See corresponding supplemental video "SupVideo2-SPc512wk11.mp4.</u> **Panel C**: Comparison of two dy2J/dy2J mice (left earing; right earing, torn) treated with AAV$_9$-CBh-αLNNdΔG2' (low dose) with an untreated dy^{2J}/dy^{2J} mouse at 11 weeks. Ambulation of treated mouse was similar to WT mice as with the higher dose treatment. The un-treated dystrophic mouse exhibited hindlimb extension contracture with impeded gait. <u>See cor-responding supplemental video "SupVideo3-lowCBh11wk.mp4".</u>

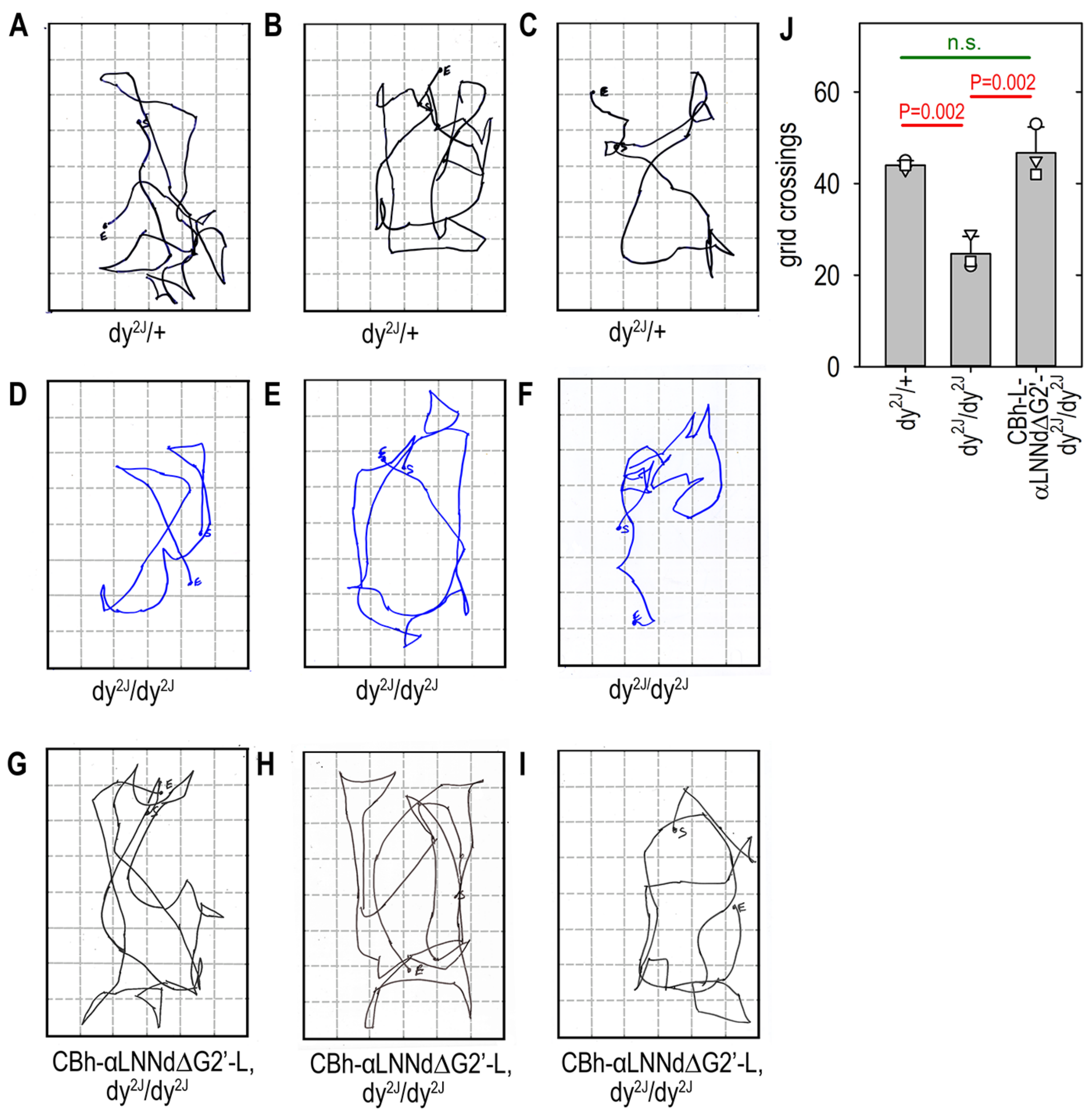

Supplemental Figure 4. *Mouse Movement Plots.* **A-I**. Paths traversed by dy^{2J}/+, dy^{2J}/dy^{2J} and AAV-CBh-αLNNdΔG2'-L (low-dose) treated dy^{2J}/dy^{2J} mice (n=3 mice/condition, 0.5 min sessions, 11 weeks age) mice (cage dimensions: top, 18.5 x 29.7 cm; base 17 x 28 cm; depth 12.5 cm) were manually recorded from the sequence of video image frames. The number of times a mouse shoulder midline crossed a 6 x 8 box border (4 x 6 overlying base) was recorded and summed as an indicator of cumulative ambulation. **J**. Graph of the sum of mouse crossings (average and s.d, n= 3 mice/condition with individual mouse values) is shown. Statistical significance was determined from the average and s.d. by 1-way ANOVA followed by Holm-Sidak pairwise comparisons. The number of crossings was higher for the CBh-treated dystrophic mice compared to the untreated dystrophic mice and nearly the same as seen with of dy^{2J}/+ mice.

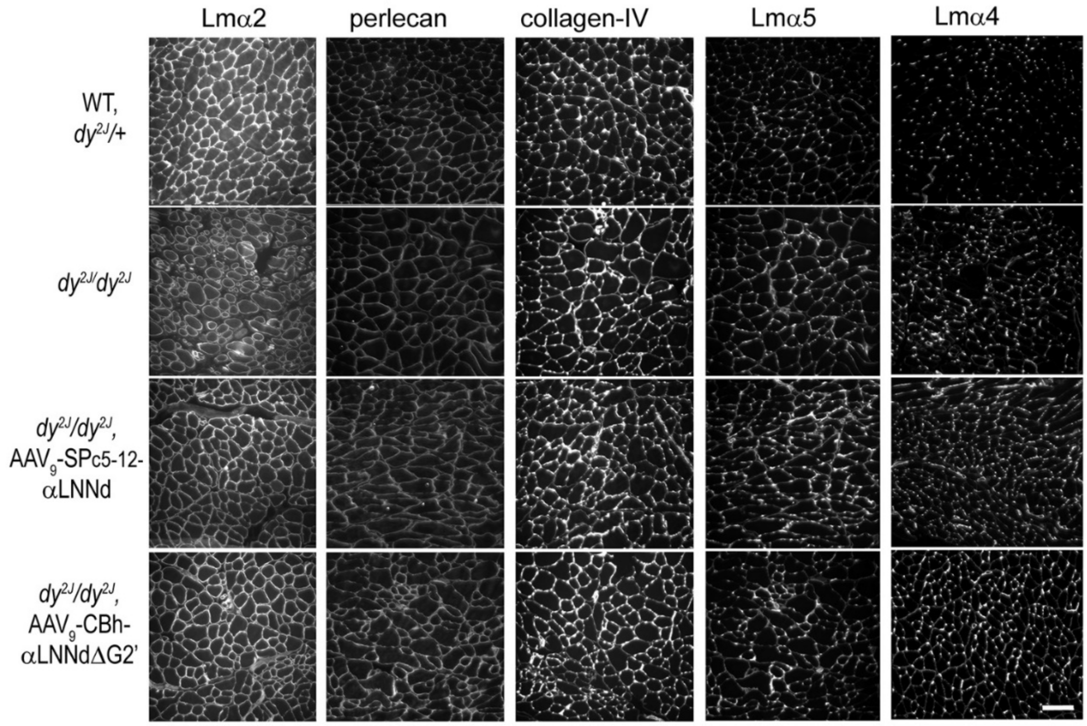

Supplemental Figure 5. *Basement membrane component immunofluorescence in skeletal muscle.* Frozen sections from lower limb muscle were immunostained to detect perlecan, collagen-IV, Lmα5 and Lmα4 in comparison with Lmα2 in dy^{2J}/+, dy^{2J}/dy^{2J} and AAV-treated dy^{2J}/dy^{2J} mice at 9 weeks of age (Bar, 100 μm). Perlecan and collagen-IV were similarly present in the BMs of all mice. Lmα4 and Lmα5 were primarily present in a microvascular distribution in dy^{2J}/+ muscle, with protein detected more strongly in a sarcolemmal pattern in untreated and treated dystrophic mice. Collagen-IV and perlecan levels were similar for all conditions examined, and notably similar when comparing αLNNd with αLNNdΔG2', suggesting that removal of the linker nidogen-G2 domain did not diminish assembly of these important BM components.

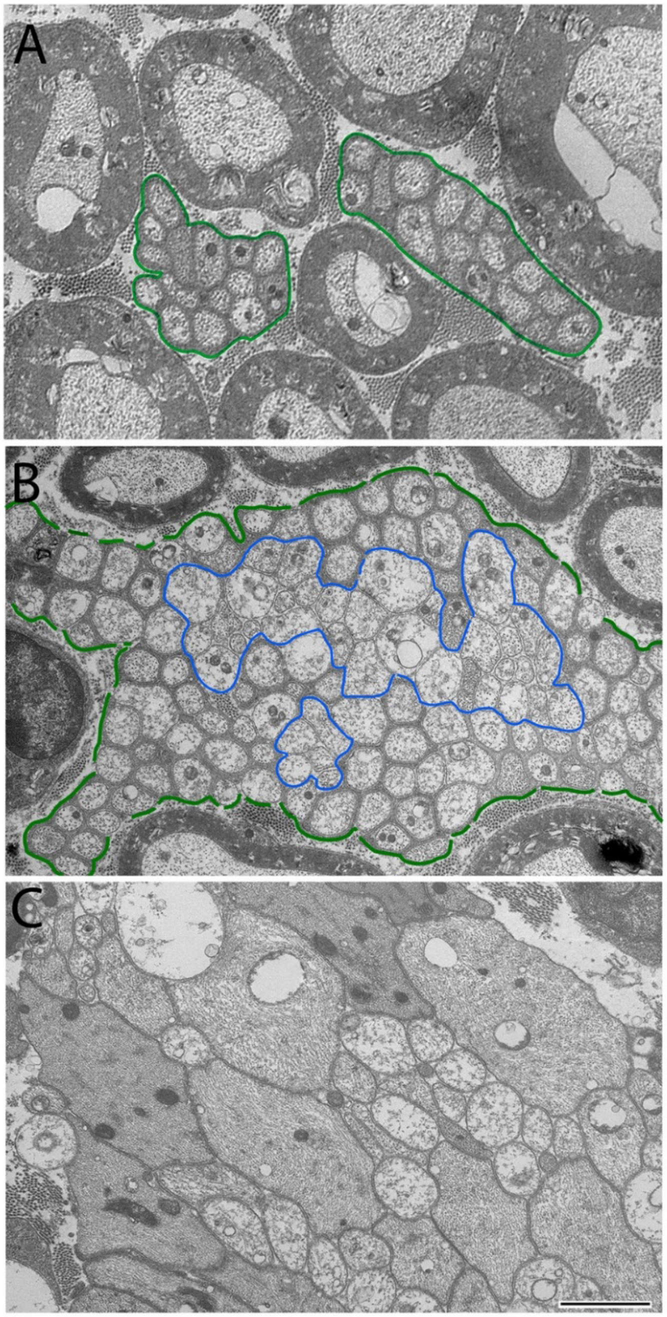

Supplemental Figure 6. *Remak bundles and amyelination patches.* **A**. Typical Remak bundle in a dy^{2J}/+ SC (Remak boundary BM marked with green curvilinear line). The axonal membranes are covered by a second SC-derived membrane and separated by darker-stained SC cytoplasm, reflecting their envelopment. **B**. Remak bundle in a dy^{2J}/dy^{2J} nerve. The SC-contained bundle, unusually large in size, is delimited by a discontinuous BM (green) and contains a large number of small caliber axons that are either enveloped or naked (naked groups delineated by blue line). **C**. Amyelination patch. The patch lies outside of a SC, has no intervening SC cytoplasm or out BM, and contains many naked axons of variable caliber, some quite large. Length bar, 2 μm.

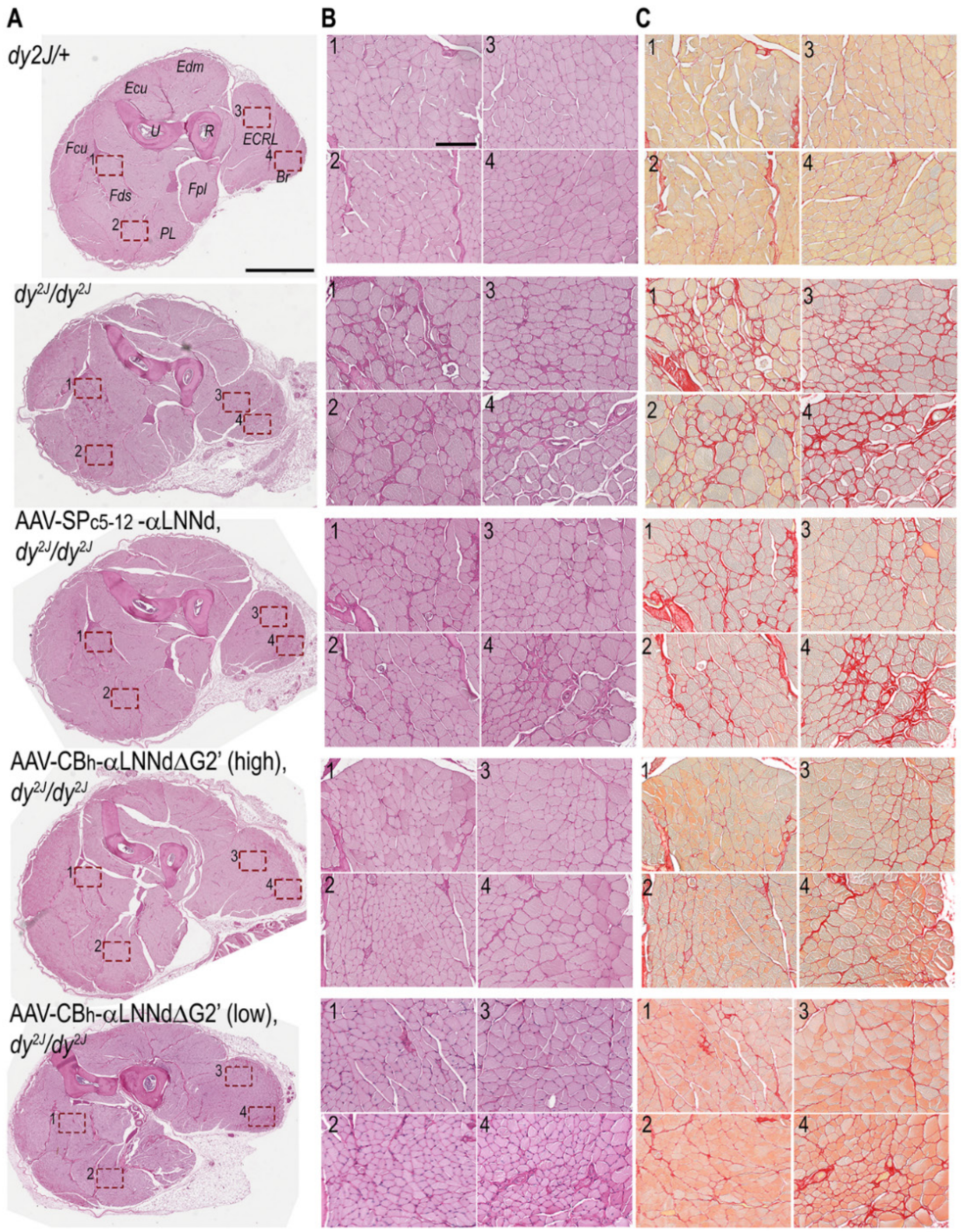

Supplemental Figure 7. *Survey of cross-section micrographs of distal forelimb muscles.* Mouse tissues were obtained from *dy*^{2J}/+, *dy*^{2J}/*dy*^{2J}, AAV-SPc5-12 αLNNd treated *dy*^{2J}/*dy*^{2J}, and AAV-CBh-αLNNdΔG2' treated *dy*^{2J}/*dy*^{2J} mice at 15 weeks age. Adjacent sections were stained with PAS (columns A, B) and PSR (column C). Boxed regions of the low magnification forelimb cross-sections are shown as 6x insets (abbreviations: U, ulna; R, radius; ECLR, extensor carpi radialis longus; Br, brachioradialis; Fpl, flexor pollicis longus; PL, palmaris longus; Fds, flexor dig. superficialis; Fcu, flexor carpi ulnaris; Ecu, flexor carpi ulnaris; Edm, ext. dig. minimi). Length bars: A, 1 mm; B, C, 100 μm. Regions of more prominent dystrophic change (rounded myofibers of variable size), accompanied by increased fibrosis (PSR stained collagen), include ECRL and Br. Treatment with the muscle-specific αLNNd partially ameliorated the pathology for boxes 1 - 3. In contrast, a considerably greater degree of amelioration was observed for all boxed regions by treatment with both high and low-dose AAV_9-CBh-αLNNdΔG2' treated *dy*^{2J}/*dy*^{2J}.

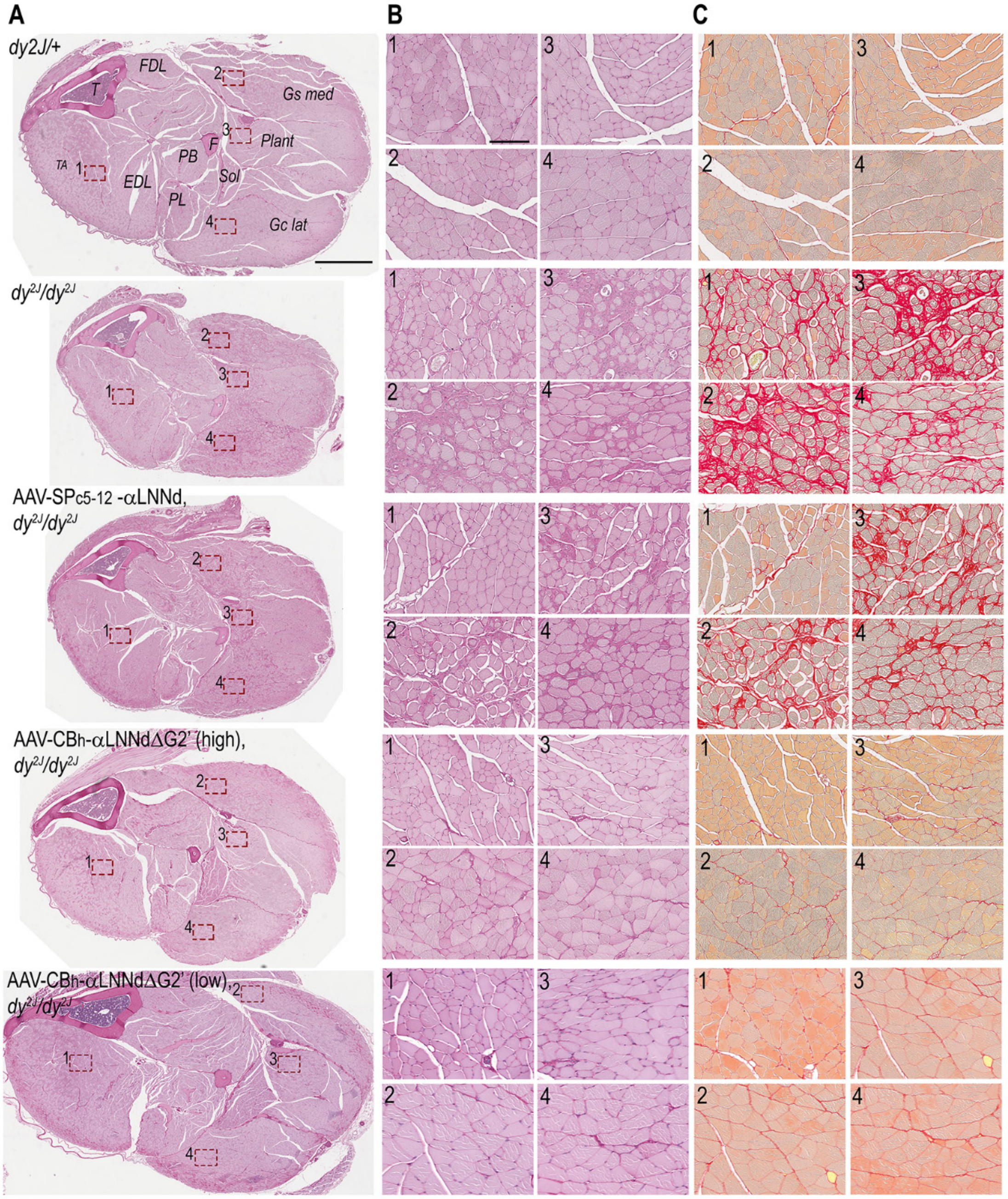

Supplemental Figure 8. *Survey of cross-section micrographs of distal hindlimb muscles.* Tissue was prepared and stained from15 week mice as in previous supplemental figure. Adjacent paraffin-embedded sections were cut from distal hindlimb and stained with PAS (A, B) and PSR (C). Box regions of the low magnification forelimb cross-sections are shown as 7x insets. Abbreviations: T, tibia; F, fibula; TA, tibialis anterior; EDL, extensor dig. longus; FDL, flexor dig. longus; PB, peroneus brevis; PL, peroneus longus; Sol, soleus; Plan, plantaris; Gs lat, gastrocnemius lateralis; Gs med, gastrocnemius medialis. Length bars: A, 1 mm; B,C, 100 μm. Regions of more prominent dystrophic change in *dy^{2J}/dy^{2J}* (rounded myofibers of variable size), accompanied by increased fibrosis (PSR stained collagen), were unevenly distributed (e.g. boxed regions), most prominently in the lateral compartment. Greater improvement of dystrophic foci, including reduced fibrosis, was observed in dystrophic muscle treated with CBh-αLNNdΔG2'.

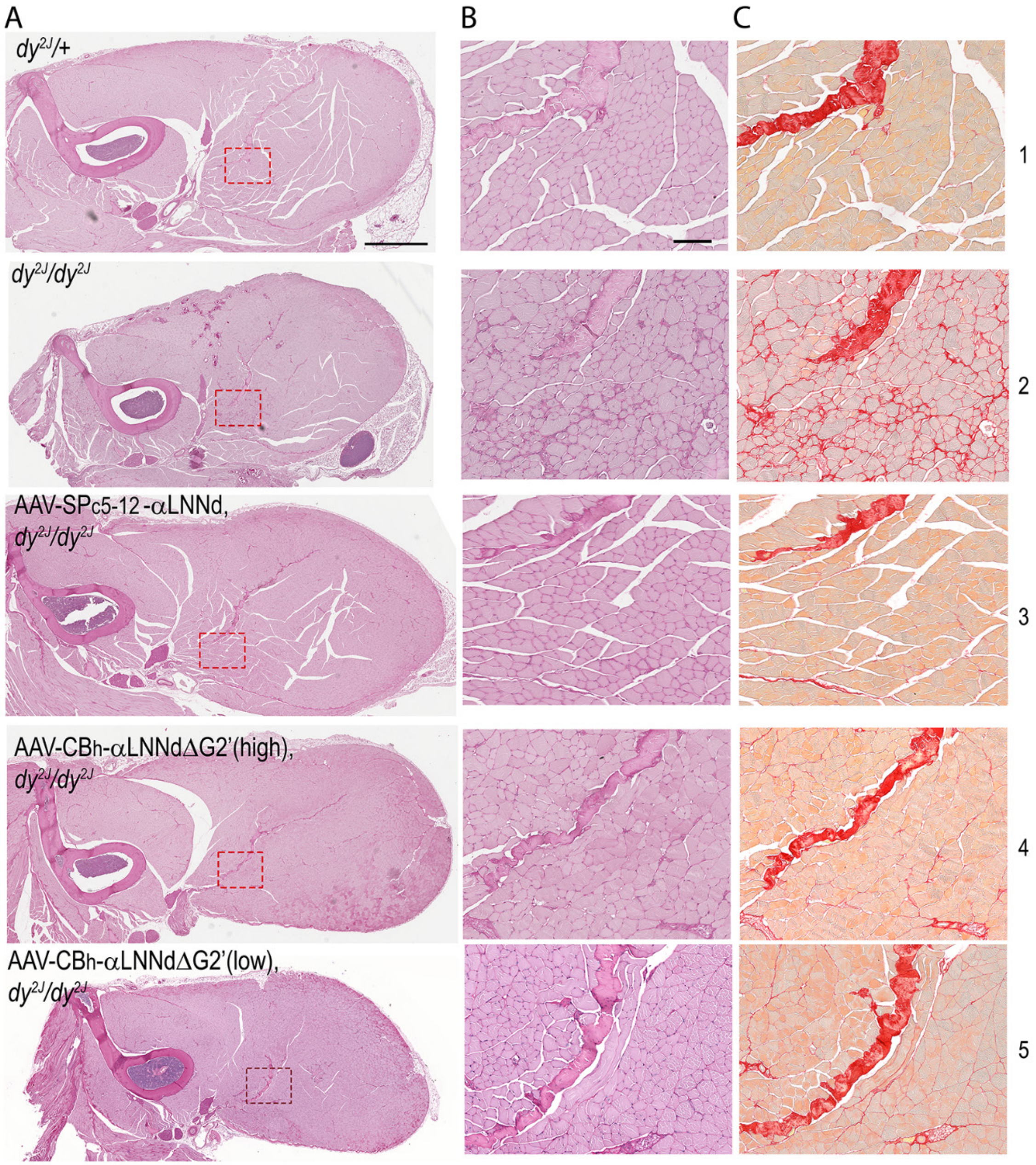

Supplemental Figure 9. *Survey of cross-section micrographs of proximal forelimb muscles*. Tissue was prepared and stained from 15-week mice as in previous supplemental figure (bar for column A panels, 1 mm; bar for magnified triceps inserts in columns B and C, 100 μm). Adjacent sections were cut from proximal forelimb and stained with either PAS (A,B) or PSR (C). Length bars: A, 1 mm; B,C, 100 μm. Proximal forelimb muscles were less affected compared to those in distal forelimb. A region of more prominent dystrophic changes is boxed in triceps and shown at higher (6x) magnification, PAS and PSR stained. Greater improvement of overall histology, and particularly of the dystrophic foci, including reduced fibrosis, was observed in the dy^{2J}/dy^{2J} muscle treated with AAV-CBh-αLNNdΔG2'.

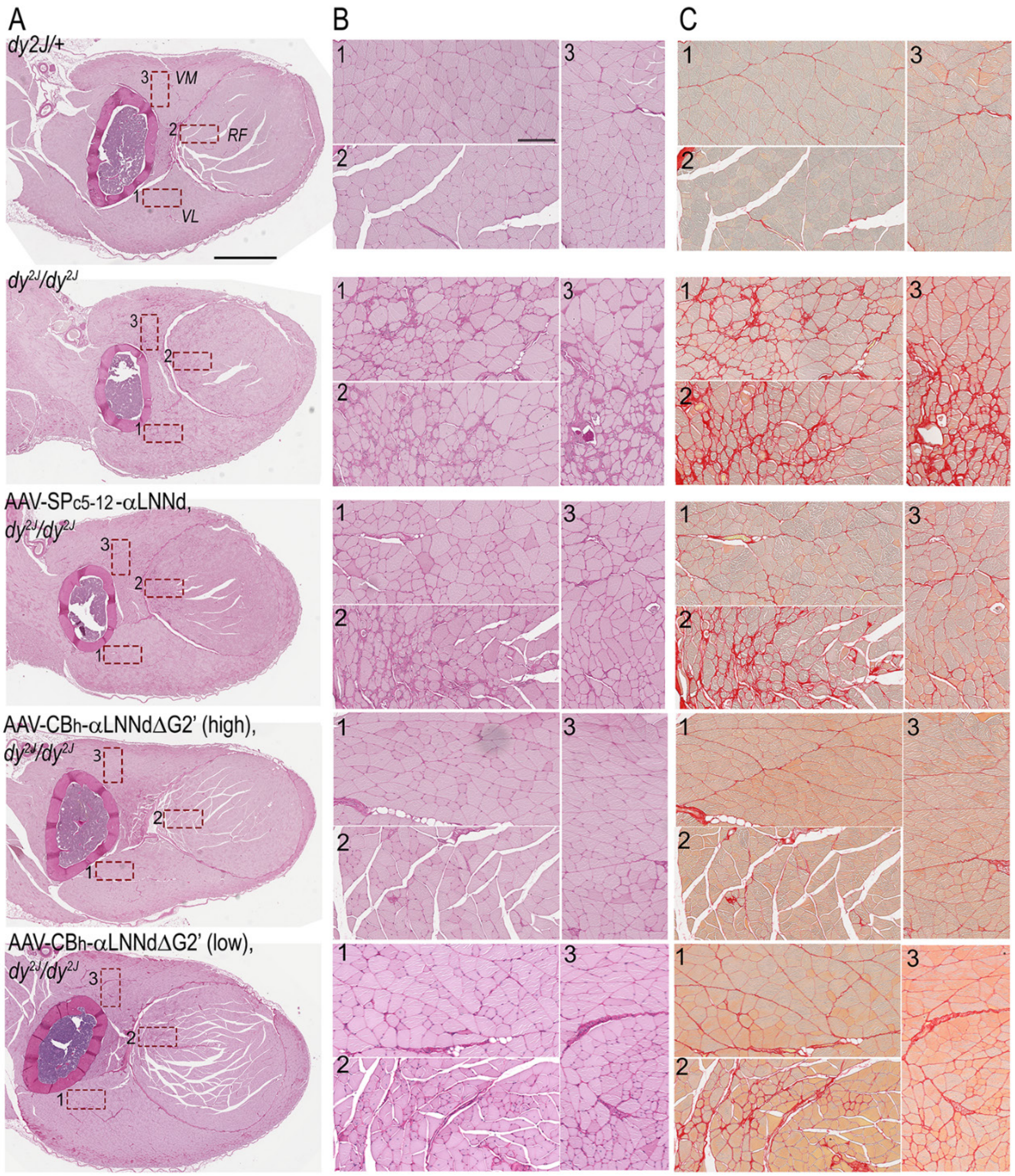

Supplemental Figure 10. *Survey of cross-section micrographs of proximal hindlimb muscles.* Tissue was prepared and stained from 15-week mice as in previous supplemental figures. Adjacent sections from proximal hindlimb and stained with either PAS (columns A, B) or PSR (column C) are shown. These muscles were less affected compared to those in distal hindlimb. Abbreviations: RF, rectus femoris; VM and VL, vastus medialis and lateralis. Size bars: A, 1 mm; B,C, 100 µm. Three regions of more prominent dystrophic changes are boxed and shown at higher (6x) magnification, PAS and PSR stained. Muscle adjacent to the femur exhibited more prominent dystrophic changes in dy^{2J}/dy^{2J}. Greater improvement of the dystrophic focus, including reduced fibrosis, was observed in dystrophic muscle treated with CBh-αLNNdΔG2'.

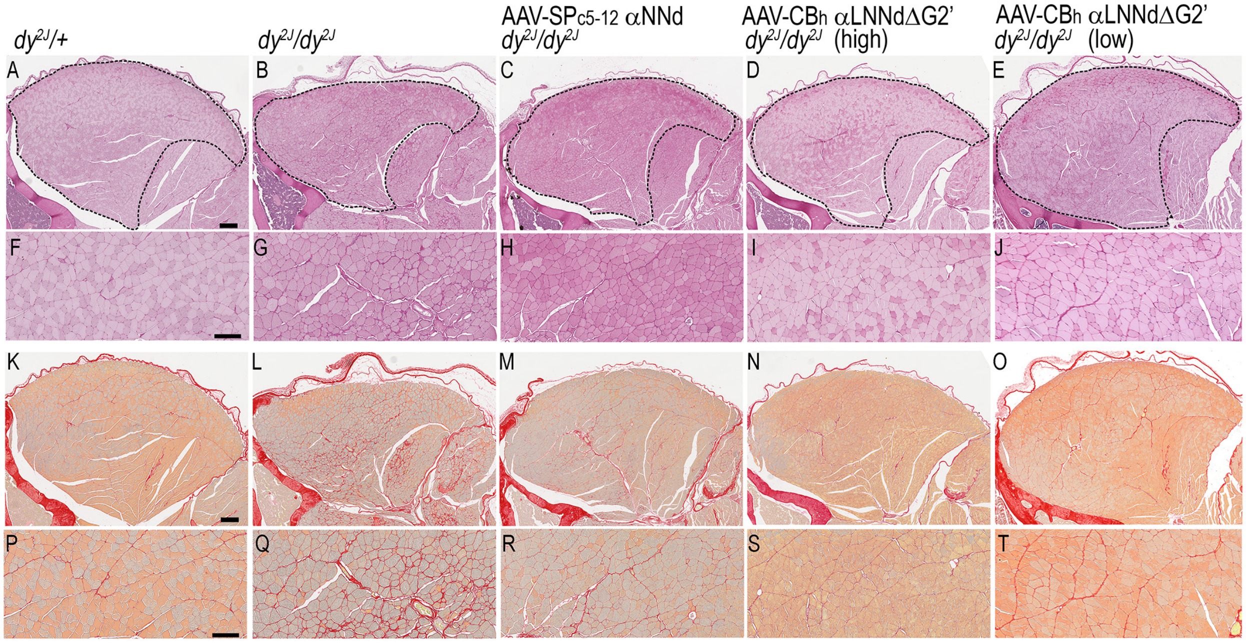

Supplemental Figure 11. *Histology of Tibialis Anterior.* Hindlimb from 15 week old mice *(dy*^{2J}/+ (panel A-D, first column), *dy*^{2J}/*dy*^{2J} (second column), and *dy*^{2J}/*dy*^{2J} treated with either AAV-αLNNd (SPc5-12 promoter, third column) or AAV- αLNNdΔG2' (CBh promoter, fourth column), was fixed, and stained with PAS (panels A and 3x detail, B) and PSR (panels C and 3x detail, D). Length bars: 200 μm for A-B and K-O and 100 μm for F-J and P-T. TA muscle exhibited modest dystrophic changes in *dy*^{2J}/*dy*^{2J} (focal rounding of myofibers, peri-myofibril fibrosis, increased fraction of central nuclei). These changed were substantially improved by all AAV-treatments.

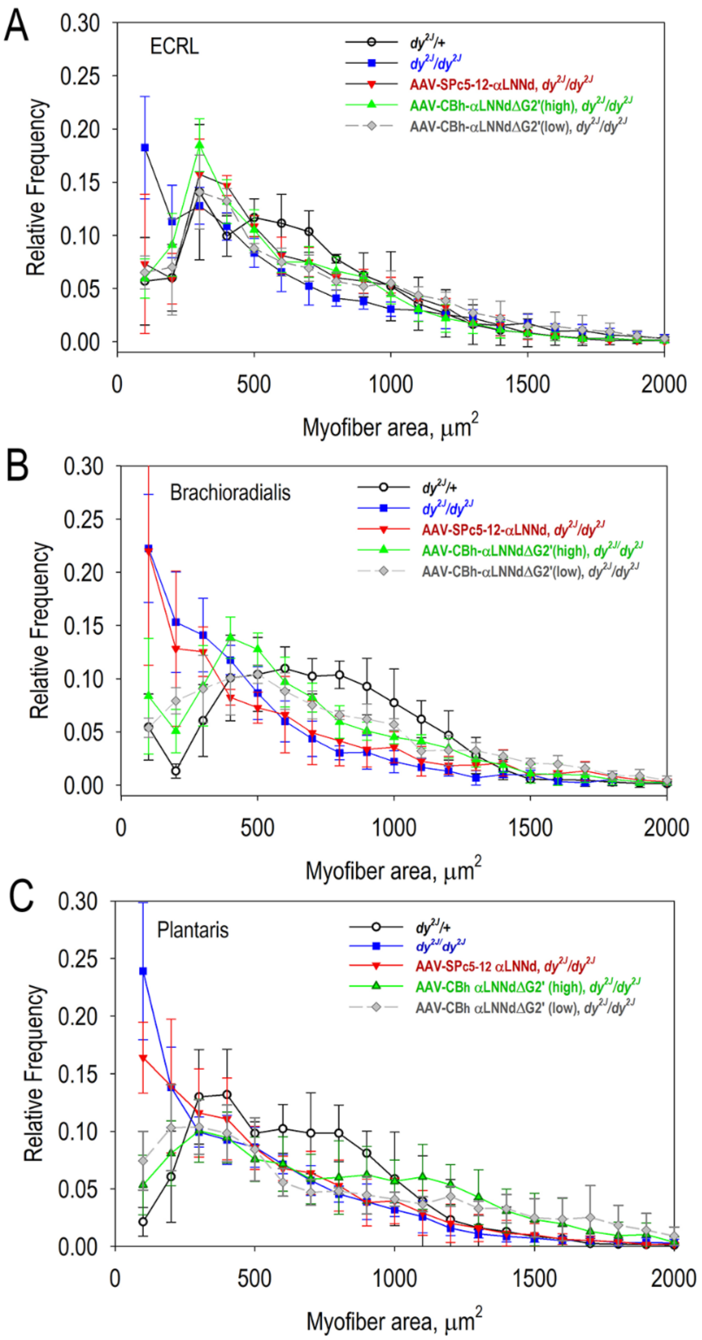

Supplemental Figure 12. Muscle histograms. Distribution of myofiber cross-section areas (average and s.d.) for extensor carpi radialis longus (A), brachioradialis (B), and plantaris (C) from *dy*^{2J}/+ (n=5), *dy*^{2J}/*dy*^{2J} (n=6), SPc5-12-αLNNd *dy*^{2J}/*dy*^{2J} (n=5), AAV-CBh-αLNNdΔG2' (high dose) *dy*^{2J}/*dy*^{2J} (n=5), and CBh-αLNNdΔG2' (low dose) *dy*^{2J}/*dy*^{2J} (n=3) of 15 week-old mice. Statistical significance was determined from the average and s.d. by 1-way ANOVA followed by Holm-Sidak test pairwise comparisons. Both AAV treatments increased the myofiber areas to values between that of untreated *dy*^{2J}/*dy*^{2J} and *dy*^{2J}/+. In brachioradialis and plantaris, AAV-CBh-αLNNdΔG2' sizes were increased beyond those of SPc5-12-αLNNd *dy*^{2J}/*dy*^{2J}.

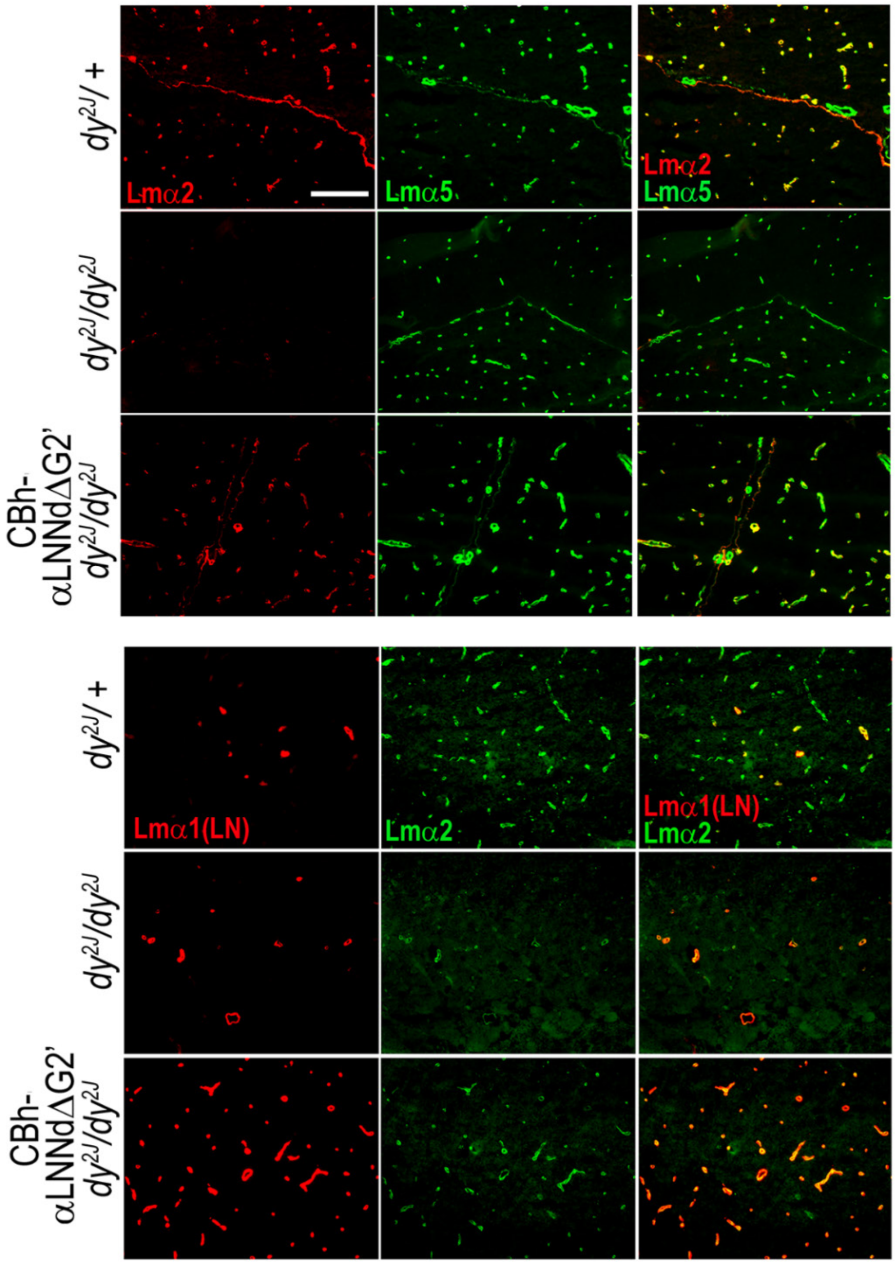

Supplemental Figure 13. ***Laminin immunofluorescence in brain cortex***. Lm-α1 (1μg/ml rabbit Ab), Lmα2 (N-terminal; rat, 1:100) and Lmα5 (chick. Ab, 1 μg/ml) were detected in capillaries, small vessels and pia (linear structures) of 11-week-old WT and dystrophic mouse brain. Length bar, 100 μm. While Lmα2 was strongly expressed in *dy*^{2J}/+, it was only weakly present in untreated *dy*^{2J}/*dy*^{2J} brain. Lmα2 brightness was increased in *dy*^{2J}/*dy*^{2J} treated with AAV9-CBh-αLNNdΔG2' (high dose). Lmα1LN was expressed in a subset of *dy*^{2J}/+ and *dy*^{2J}/*dy*^{2J} capillaries and widely expressed in capillaries of AAV-treated *dy*^{2J}/*dy*^{2J}**.** The increase in the Lmα2 subunit is attributed to the accumulation of linker protein.

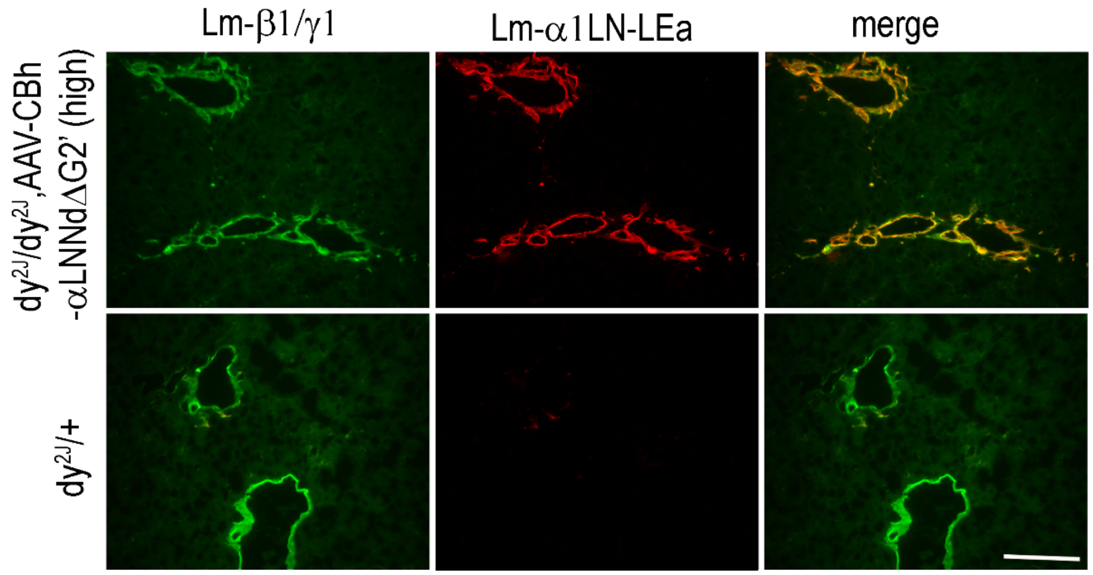

Supplemental Figure 14. *Liver expression of linker protein.* Liver from 11-week-old *dy*^{2J}/+ and *dy*^{2J}/*dy*^{2J} mice treated with AAV-CBh-αLNNdΔG2' (high dose) were examined by immunostaining for laminin (β1/γ1 chain antibody) and linker protein (Lm-α1LN-LEa specific antibody). Length bar, 100 μm. Linker protein expression was detected only in the BMs of vessels and bile ducts.

Supplemental Table I. ***AAV9 Constructs for Linker Protein Transgene Expression***

DNA element:	*αLNNd*	*αLNNdΔG2'*
5'ITR	141	141
intervening	33	55
SPc5-12 promoter	383	
CBh promoter		798
intervening		25
Kozak	6	6
αLNNd	4143	
αLNNdΔG2'		2997
WPRE		598
poly(A) signal	49	49
intervening	19	85
3' ITR	141	141
TOTAL	*4915*	*4895*

Construct design. To accommodate full-length αLNNd within a 5 kB capsid limit and a short poly(A) tail of 49 bp, the promoter could be no larger than 468 bp (at this upper limit expression levels tend to be low). Small ubiquitous promoters falling within this size constraint are PGK (426 bp), UBC (403 bp), and GUSB (378 bp) (1, 2). However, these promoters drive weak expression (1, 2). SPc5-12, on the other hand, is a synthetic muscle-specific promoter reported to provide high expression (six- to eight-fold stronger than the CMV promoter in skeletal muscle) (3). It is thus sufficiently small for expression of full-length αLNNd. However, a ubiquitous promoter is needed if one is to obtain nerve as well as muscle expression, affected in LAMA2-RDs. While a non-proprietary high-expression small ubiquitous promoter was not identified, it was found that the DNA coding for the αLN-linker protein could be reduced in size to 2997 bp with retention of the essential binding activities, providing room for as much as 1614 bp. CBh is a suitably-size (798 bp) promoter reported to provide higher expression compared to CMV and CBA (4), leaving room for addition of a post-transcriptional regulatory element (WPRE) to further increase and stabilize expression.

References:

1. Haery L, Deverman BE, Matho KS, Cetin A, Woodard K, Cepko C, et al. Adeno-Associated Virus Technologies and Methods for Targeted Neuronal Manipulation. *Front Neuroanat.* 2019;13:93.
2. Powell SK, Rivera-Soto R, and Gray SJ. Viral expression cassette elements to enhance transgene target specificity and expression in gene therapy. *Discov Med.* 2015;19(102):49-57.
3. Li X, Eastman EM, Schwartz RJ, and Draghia-Akli R. Synthetic muscle promoters: activities exceeding naturally occurring regulatory sequences. *Nat Biotechnol.* 1999;17(3):241-5.
4. Gray SJ, Foti SB, Schwartz JW, Bachaboina L, Taylor-Blake B, Coleman J, et al. Optimizing promoters for recombinant adeno-associated virus-mediated gene expression in the peripheral and central nervous system using self-complementary vectors. *Hum Gene Ther.* 2011;22(9):1143-53.

Supplemental Table II. ***Grip-strengths. Significance determined by one-way ANOVA followed by Holm-Sidak pairwise comparisons.*** Genotype/AAV codes: **#1** = WT; **#2** = AAV$_9$-CB$_h$-αLNNdΔG2' ,high dose; **#3** = *dy*^{2J}/*dy*^{2J}; **#4** = AAV$_9$-SPc5-12-αLNNd in *dy*^{2J}/*dy*^{2J}. **#5** = AAV$_9$-CBh-αLNNdΔG2', low dose. P-values (N.S., not significant).

wks	limb	#1 vs. #3	#1 vs. #2	#2 vs. #3	#1 vs. #4	2 vs. #4	#3 vs. #4	#1 vs. #5	#2 vs. #5	#3 vs. #5	#4 vs. #5	mouse count for ea. type				
												#1	*#2*	*#3*	*#4*	*#5*
3	forelimb	0.042	N.S.	N.S.	N.S.	N.S.	N.S.	N.S.	N.S.	N.S.	N.S.	22	9	9	9	7
4	forelimb	<0.001	N.S.	<0.001	0.001	N.S.	<0.001	<0.001	N.S.	<0.001	N.S.	24	9	11	9	7
5	forelimb	<0.001	0.021	<0.001	0.001	N.S.	<0.001	0.001	N.S.	<0.001	N.S.	24	9	11	9	7
6	forelimb	<0.001	0.014	<0.001	0.007	N.S.	<0.001	<0.001	N.S.	<0.001	N.S.	24	9	11	9	7
7	forelimb	<0.001	N.S.	<0.001	0.001	N.S.	<0.001	0.013	N.S.	<0.001	N.S.	23	9	11	9	7
8	forelimb	<0.001	N.S.	<0.001	0.001	N.S.	<0.001	0.002	N.S.	<0.001	N.S.	22	9	11	9	7
9	forelimb	<0.001	N.S.	<0.001	N.S.	N.S.	<0.001	<0.001	N.S.	0.019	N.S.	18	9	11	9	7
10	forelimb	<0.001	N.S.	<0.001	N.S.	N.S.	<0.001	0.012	N.S.	0.004	N.S.	15	8	10	7	7
11	forelimb	<0.001	N.S.	<0.001	N.S.	N.S.	0.002	0.006	N.S.	N.S.	N.S.	14	8	8	7	7
15	forelimb	<0.001	N.S.	0.011	N.S.	N.S.	0.008	N.S.	N.S.	N.S.	0.083	12	7	5	7	7
wks	limb	#1 vs. #3	#1 vs. #2	#2 vs. #3	#1 vs. #4	2 vs. #4	#3 vs. #4	#1 vs. #5	#2 vs. #5	#3 vs. #5	#4 vs. #5	*#1*	*#2*	*#3*	*#4*	*#5*
3	hindlimb	N.S.	N.S.	N.S.	N.S.	N.S.	N.S.	N.S.	N.S.	N.S.	N.S.	22	9	9	9	7
4	hindlimb	<0.001	N.S.	<0.001	<0.001	<0.001	N.S.	0.017	0.005	N.S.	N.S.	24	9	11	9	7
5	hindlimb	<0.001	N.S.	<0.001	<0.001	0.010	N.S.	<0.001	0.008	N.S.	N.S.	24	9	11	9	7
6	hindlimb	<0.001	N.S.	<0.001	0.003	N.S.	0.005	0.001	N.S.	0.049	N.S.	24	9	11	9	7
7	hindlimb	<0.001	N.S.	<0.001	<0.001	<0.001	N.S.	<0.001	0.001	0.030	N.S.	23	9	11	9	7
8	hindlimb	<0.001	0.043	<0.001	<0.001	0.005	N.S.	<0.001	N.S.	0.014	N.S.	22	9	11	9	7
9	hindlimb	<0.001	N.S.	<0.001	<0.001	<0.001	N.S.	<0.001	0.011	0.006	N.S.	18	9	11	9	7
10	hindlimb	<0.001	N.S.	<0.001	<0.001	<0.001	N.S.	0.003	N.S.	<0.001	0.021	15	8	10	7	7
11	hindlimb	<0.001	N.S.	<0.001	<0.001	0.007	N.S.	0.007	N.S.	0.007	N.S.	14	8	8	7	7
15	hindlimb	<0.001	N.S.	<0.001	<0.001	0.002	N.S.	0.010	N.S.	0.004	N.S.	12	7	5	7	7
wks	limb:	#1 vs. #3	#1 vs. #2	#2 vs. #3	#1 vs. #4	2 vs. #4	#3 vs. #4	#1 vs. #5	#2 vs. #5	#3 vs. #5	#4 vs. #5	*#1*	*#2*	*#3*	*#4*	*#5*
3	all limbs	<0.001	N.S.	<0.001	<0.001	0.006	N.S.	N.S.	N.S.	<0.001	N.S.	22	9	9	9	7
4	all limbs	<0.001	N.S.	<0.001	<0.001	0.002	0.003	N.S.	N.S.	<0.001	N.S.	24	9	11	9	7
5	all limbs	<0.001	N.S.	<0.001	<0.001	0.011	0.002	0.003	0.049	<0.001	N.S.	24	9	11	9	7
6	all limbs	<0.001	N.S.	<0.001	<0.001	0.015	0.001	0.006	N.S.	<0.001	N.S.	24	9	11	9	7
7	all limbs	<0.001	N.S.	<0.001	<0.001	<0.001	0.019	<0.001	0.028	<0.001	0.002	23	9	11	9	7
8	all limbs	<0.001	N.S.	<0.001	<0.001	<0.001	N.S.	0.001	N.S	<0.001	N.S.	22	9	11	9	7
9	all limbs	<0.001	N.S.	<0.001	<0.001	<0.001	0.015	<0.001	0.004	0.004	N.S.	18	9	11	9	7
10	all limbs	<0.001	N.S.	<0.001	<0.001	<0.001	0.006	0.013	0.022	<0.001	0.008	15	8	10	7	7
11	all limbs	<0.001	N.S.	<0.001	<0.001	0.001	N.S.	0.045	N.S.	<0.001	N.S.	14	8	8	7	7
15	all limbs	<0.001	N.S.	<0.001	<0.001	0.011	N.S.	0.005	N.S.	N.S.	N.S.	12	7	5	7	7
